# Malaria Surveillance — United States, 2018

**DOI:** 10.15585/mmwr.ss7108a1

**Published:** 2022-09-02

**Authors:** Kimberly E. Mace, Naomi W. Lucchi, Kathrine R. Tan

**Affiliations:** 1 Malaria Branch, Division of Parasitic Diseases and Malaria, Center for Global Health, CDC

## Abstract

**Problem/Condition:**

Malaria in humans is caused by intraerythrocytic protozoa of the genus *Plasmodium*. These parasites are transmitted by the bite of an infective female *Anopheles* species mosquito. Most malaria infections in the United States and its territories occur among persons who have traveled to regions with ongoing malaria transmission. However, among persons who have not traveled out of the country, malaria is occasionally acquired through exposure to infected blood or tissues, congenital transmission, nosocomial exposure, or local mosquitoborne transmission. Malaria surveillance in the United States and its territories provides information on its occurrence (e.g., temporal, geographic, and demographic), guides prevention and treatment recommendations for travelers and patients, and facilitates rapid transmission control measures if locally acquired cases are identified.

**Period Covered:**

This report summarizes confirmed malaria cases in persons with onset of illness in 2018 and trends in previous years.

**Description of System:**

Malaria cases diagnosed by blood smear microscopy, polymerase chain reaction, or rapid diagnostic tests are reported to local and state health departments through electronic laboratory reports or by health care providers or laboratory staff members directly reporting to CDC or health departments. Case investigations are conducted by local and state health departments, and reports are transmitted to CDC through the National Malaria Surveillance System (NMSS), the National Notifiable Diseases Surveillance System (NNDSS), or direct CDC clinical consultations. CDC reference laboratories provide diagnostic assistance and conduct antimalarial drug resistance marker testing on blood specimens submitted by health care providers or local or state health departments. This report summarizes data from the integration of all cases from NMSS and NNDSS, CDC clinical consultations, and CDC reference laboratory reports.

**Results:**

CDC received reports of 1,823 confirmed malaria cases with onset of symptoms in 2018, including one cryptic case and one case acquired through a bone marrow transplant. The number of cases reported in 2018 is 15.6% fewer than in 2017. The number of cases diagnosed in the United States and its territories has been increasing since the mid-1970s; the number of cases reported in 2017 was the highest since 1972. Of the cases in 2018, a total of 1,519 (85.0%) were imported cases that originated from Africa; 1,061 (69.9%) of the cases from Africa were from West Africa, a similar proportion to what was observed in 2017. Among all cases, *P. falciparum* accounted for most infections (1,273 [69.8%]), followed by *P. vivax* (173 [9.5%]), *P. ovale* (95 [5.2%]), and *P. malariae* (48 [2.6%]). For the first time since 2008, an imported case of *P. knowlesi* was identified in the United States and its territories. Infections by two or more species accounted for 17 cases (<1.0%). The infecting species was not reported or was undetermined in 216 cases (11.9%). Most patients (92.6%) had symptom onset <90 days after returning to the United States or its territories from a country with malaria transmission. Of the U.S. civilian patients who reported reason for travel, 77.0% were visiting friends and relatives. Chemoprophylaxis with antimalarial medications are recommended for U.S. residents to prevent malaria while traveling in countries where it is endemic. Fewer U.S. residents with imported malaria reported taking any malaria chemoprophylaxis in 2018 (24.5%) than in 2017 (28.4%), and adherence was poor among those who took chemoprophylaxis. Among the 864 U.S. residents with malaria for whom information on chemoprophylaxis use and travel region were known, 95.0% did not adhere to or did not take a CDC-recommended chemoprophylaxis regimen. Among 683 women with malaria, 19 reported being pregnant. Of these, 11 pregnant women were U.S. residents, and one of whom reported taking chemoprophylaxis to prevent malaria but her adherence to chemoprophylaxis was not reported. Thirty-eight (2.1%) malaria cases occurred among U.S. military personnel in 2018, more than in 2017 (26 [1.2%]). Among all reported malaria cases in 2018, a total of 251 (13.8%) were classified as severe malaria illness, and seven persons died from malaria. In 2018, CDC analyzed 106 *P. falciparum*-positive and four *P. falciparum* mixed species specimens for antimalarial resistance markers (although certain loci were untestable in some specimens); identification of genetic polymorphisms associated with resistance to pyrimethamine were found in 99 (98.0%), to sulfadoxine in 49 (49.6%), to chloroquine in 50 (45.5%), and to mefloquine in two (2.0%); no specimens tested contained a marker for atovaquone or artemisinin resistance.

**Interpretation:**

The importation of malaria reflects the overall trends in global travel to and from areas where malaria is endemic, and 15.6% fewer cases were imported in 2018 compared with 2017. Of imported cases, 59.3% were among persons who had traveled from West Africa. Among U.S. civilians, visiting friends and relatives was the most common reason for travel (77.1%).

**Public Health Actions:**

The best way for U.S. residents to prevent malaria is to take chemoprophylaxis medication before, during, and after travel to a country where malaria is endemic. Adherence to recommended malaria prevention strategies among U.S. travelers would reduce the number of imported cases. Reported reasons for nonadherence include prematurely stopping after leaving the area where malaria was endemic, forgetting to take the medication, and experiencing a side effect. Health care providers can make travelers aware of the risks posed by malaria and incorporate education to motivate them to be adherent to chemoprophylaxis. Malaria infections can be fatal if not diagnosed and treated promptly with antimalarial medications appropriate for the patient’s age, pregnancy status, medical history, the likely country of malaria acquisition, and previous use of antimalarial chemoprophylaxis. Antimalarial use for chemoprophylaxis and treatment should be determined by the CDC guidelines, which are frequently updated. In April 2019, intravenous (IV) artesunate became the first-line medication for treatment of severe malaria in the United States and its territories. Artesunate was approved by the Food and Drug Administration (FDA) in 2020 and is commercially available (Artesunate for Injection) from major U.S. drug distributors (https://amivas.com). Stocking IV artesunate locally allows for immediate treatment of severe malaria once diagnosed and provides patients with the best chance of a complete recovery and no sequelae. With commercial IV artesunate now available, CDC will discontinue distribution of non–FDA-approved IV artesunate under an investigational new drug protocol on September 30, 2022. Detailed recommendations for preventing malaria are online at https://www.cdc.gov/malaria/travelers/drugs.html. Malaria diagnosis and treatment recommendations are also available online at https://www.cdc.gov/malaria/diagnosis_treatment. Health care providers who have sought urgent infectious disease consultation and require additional assistance on diagnosis and treatment of malaria can call the Malaria Hotline 9:00 a.m.–5:00 p.m. Eastern Time, Monday–Friday, at 770-488-7788 or 855-856-4713 or after hours for urgent inquiries at 770-488-7100. Persons submitting malaria case reports (care providers, laboratories, and state and local public health officials) should provide complete information because incomplete reporting compromises case investigations and public health efforts to prevent future infections and examine trends in malaria cases. Molecular surveillance of antimalarial drug resistance markers enables CDC to track, guide treatment, and manage drug resistance in malaria parasites both domestically and globally. A greater proportion of specimens from domestic malaria cases are needed to improve the completeness of antimalarial drug resistance analysis; therefore, CDC requests that blood specimens be submitted for any case of malaria diagnosed in the United States and its territories.

## Introduction

Malaria, a febrile parasitic illness transmitted through the bite of an infective mosquito, was estimated to cause 241 million illnesses in 2020 and 627,000 deaths worldwide (*1*). In 2018, there were an estimated 228 million cases of malaria, globally, and 405,000 deaths (*2*). Malaria is transmitted in 85 countries, and approximately one half of the world population is at risk for infection (*1*). Since 2000, the global community has funded and implemented malaria control efforts and achieved an estimated 23 million fewer malaria cases in 2018 alone, compared with 2010, and cumulatively, these efforts have prevented millions of malaria deaths. However, during 2014–2018, the number of cases globally was stable (*2*), and in 2020 the number of malaria cases increased compared with previous years, in part because of disruption of health services and interventions from the COVID-19 pandemic (*1*). In October 2021, the first malaria vaccine was recommended by the World Health Organization to protect infants and children aged 5–24 months living in moderate-to-high transmission areas (*3*).

*Plasmodium* parasites that cause malaria are transmitted through the bite of an infective *Anopheles* mosquito, resulting in an intraerythrocytic illness, which can range from asymptomatic or mild to severe and fatal. Four species of *Plasmodium* cause illness in humans: *P. falciparum, P. vivax*, *P. ovale* species (caused by *P. ovale curtisi* and *P. ovale wallikeri*), and *P. malariae*. Simian malarias can also cause illness in humans, particularly *P. knowlesi* in Southeast Asia. *P. falciparum* causes the most infections worldwide; it is predominant in Africa where an estimated 95% of cases occur (*1*,*2*), and partly because of a rapid replication rate, infections from this species can rapidly progress to severe illness, especially for infants and children aged <5 years and among persons who do not have acquired immunity (*4*). In 2018, *P. vivax* accounted for approximately 3% of cases worldwide. *P. vivax* is found in a broad geographical area and although it contributed <1% of cases in Africa, it made up 75% of cases in the Americas, 50% of cases in Asia, and approximately 30%–35% of cases in the Eastern Mediterranean and Western Pacific regions (*2*). Compared with *P. vivax* and *P. falciparum*, transmissions of *P. ovale* spp., *P. malariae* and *P. knowlesi* species are limited. Approximately 95% of *P. ovale* spp. cases were identified in Africa, with 5% in Asia (*5*). *P. vivax* and *P. ovale* parasites have a dormant stage (hypnozoite) in the liver, making relapse common during the period of 45 days to 3 years after an initial illness (*4*). *P. vivax* parasites transmitted in some areas of Asia can have a long incubation period, lasting 6 or more months from inoculation to symptom onset (*6*–*8*). *P. malariae* parasites are found throughout the tropics and subtropics and are often detected in mixed species infections. *P. malariae* parasites mature slowly in human and mosquito hosts and, although they do not typically cause severe symptoms in humans, can result in persistent low-density infections that can last for years, providing opportunities for ongoing transmission and health sequelae (*4*,*9*). *P. knowlesi* is predominantly a simian malaria found in Southeast Asia; however, it can be transmitted to persons, and in Malaysia, it has become the predominant species that causes malaria illness in humans (*10*–*12*). Exposure to forested areas with simian habitat is a risk factor for *P. knowlesi*, and these infections can be serious or fatal (*10*,*13*).

Although malaria was eliminated from the United States* in the mid-1950s (*14*), the *Anopheles* mosquito vector still exists throughout the United States (*15*). Since 1957, malaria surveillance has been supported to detect cases and prevent reintroduction, monitor antimalarial resistance, assess trends in case acquisition, and guide malaria prevention and treatment recommendations for U.S. residents. Most malaria cases diagnosed in the United States are imported from countries with ongoing mosquitoborne transmission. Occasionally, congenitally acquired cases, induced cases (resulting from exposure to blood or tissue products), and cryptic cases (for which exposure cannot be easily explained despite investigation by state and local health departments and CDC) occur. During 1957–2003, a total of 63 malaria outbreaks occurred in the United States. The last well-documented local mosquitoborne transmission occurred in 2003, when eight cases were diagnosed among nontravelers in Palm Beach, Florida (*16*–*18*).

Clinical illness results from the presence of an asexual, intraerythrocytic stage of the parasite in red blood cells. Factors that contribute to variability in illness severity are complex and include the parasite species and density of infection, the patient’s age and immune response to the infection, the presence of acquired or protective immunity, the patient’s general health and nutritional constitution, chemoprophylaxis effects, and time to initiate appropriate treatment (*4*). Persons that live in areas with high malaria transmission who experience repeated malarial illnesses might develop partial protective immunity that can result in less severe illness or even asymptomatic parasitemia. However, without continual exposure, this semi-immunity will be lost within a few years (*19*–*21*), and thus it is assumed that U.S. residents do not have any degree of protective immunity to malaria and are susceptible to severe illness and death. Although malaria symptoms vary by age and immunologic status, fever is the predominant symptom (*22*). Symptoms associated with uncomplicated malaria include chills, sweating, headache, fatigue, myalgia, cough, nausea, and mild anemia. If not treated promptly, malaria can rapidly progress and affect multiple organ systems and result in altered consciousness (cerebral malaria), seizures, severe anemia, acute kidney injury, liver failure, respiratory distress, coma, permanent disability, and death. Travel history should be routinely requested for patients with fever. All persons who have fever and who recently traveled to areas where malaria is endemic as well as persons who have unexplained fever, regardless of travel history, should be tested immediately for malaria.

To prevent malaria, CDC recommends that U.S. residents use chemoprophylaxis (i.e., antimalarial medication taken before, during, and after travel to a country with malaria transmission). Persons who intend to travel should ask their physician for a prescription for an antimalarial that is appropriate for the country or region of travel, the age of the patient, pregnancy status, and individual preferences (e.g., cost or regimen type [daily versus weekly]). CDC provides chemoprophylaxis guidelines to health care providers and the public (Table 1). Implementing mosquito avoidance measures provides additional protection and includes the use of repellents, use of permethrin-treated clothing, sleeping in screened sleep spaces, and using an insecticide-treated bed net (*23*).

**TABLE 1:** Resources for malaria chemoprophylaxis, diagnosis, and treatment guidelines

Subject	Source	Availability	Contact information
**Chemoprophylaxis**	CDC Traveler's Health internet site (includes online access to *Health Information for International Travel*)	24 hours/day	http://wwwnc.cdc.gov/travel
	*Health Information for International Travel (Yellow Book)*	The latest edition is available for sale from Oxford University Press (https://global.oup.com/academic/?cc=us&lang=en&) and from major online booksellers	https://wwwnc.cdc.gov/travel/page/yellowbook-home-2020; 800-445-9714; custserv.us@oup.com; http://www.oup.com/us
	CDC Malaria Branch website with malaria and chemoprophylaxis information by country	24 hours/day	http://www.cdc.gov/malaria/travelers/country_table/a.html
**Diagnosis**	CDC Division of Parasitic Diseases and Malaria diagnostic Internet site (DPDx)	24 hours/day	https://www.cdc.gov/dpdx/index.html
	CDC Division of Parasitic Diseases and Malaria diagnostic Internet site (DPDx): Bench Aids	24 hours/day	https://www.cdc.gov/dpdx/diagnosticprocedures/index.html
**Treatment**	CDC malaria treatment guidelines	24 hours/day	https://www.cdc.gov/malaria/diagnosis_treatment/clinicians1.html
**Clinical advice**	CDC Malaria Hotline ***For clinicians and blood banks only***	9:00 a.m.–5:00 p.m. Eastern Time, Monday–Friday	770-488-7788 or 855-856-4713
		After hours, on weekends, and on holidays	770-488-7100
**Malaria questions**	CDC Information ***For the general public***	8:00 a.m.–8:00 p.m. Eastern Time, Monday–Friday	1-800-CDC-INFO (1-800-232-4636)
		24 hours/day	https://wwwn.cdc.gov/dcs/ContactUs/Form

This report summarizes malaria cases reported to CDC with onset of symptoms in 2018, describes trends during previous years, and highlights information on risk factors and prevention. The intended audience includes public health authorities, health care providers, and persons traveling to areas with malaria transmission. Information on chemoprophylaxis, diagnosis, and treatment is provided for health care professionals and the public, and links to additional malaria information and resources are provided.

## Methods

### Data Sources and Analysis

Malaria case reports were submitted to CDC through the National Malaria Surveillance System (NMSS) and the National Notifiable Diseases Surveillance System (NNDSS) (*24*). The Armed Forces Health Surveillance Division provides reports of malaria among military personnel to NMSS. As a notifiable condition, positive malaria laboratory test results are automatically reported from hospital, commercial, public health, and other laboratories to state and local health departments through the electronic laboratory reporting system (*25*). The electronic laboratory reports prompt investigations by state and local health departments that are submitted to CDC via NNDSS and NMSS (*26*). Both systems rely on passive reporting from the jurisdictions, and the number of cases might differ (e.g., because of differences in date classifications). NNDSS report dates might be assigned according to the date of diagnosis or the date reported to the health department, and NMSS assigns dates according to illness onset. In addition, the NNDSS reporting system provides only basic case demographic information, whereas NMSS collects detailed epidemiologic data, including laboratory results, travel history, and clinical history, which facilitate investigation and classification of each case. Certain cases are reported through direct consultation with CDC staff members via the Malaria Hotline. Diagnostic confirmation of cases often is facilitated by the CDC reference laboratory. This report summarizes data from the integration of all NMSS and NNDSS cases and CDC reference laboratory reports after deduplication and reconciliation. This activity was reviewed by CDC and was conducted consistent with applicable federal law and CDC policy.^†^

Malaria cases are classified as confirmed or suspected using the 2014 case definition from the Council of State and Territorial Epidemiologists and CDC (*27*). Malaria cases are further categorized by infecting *Plasmodium* species. When more than a single species is detected, the case is categorized as a mixed infection. All categories are mutually exclusive. A confirmed case is diagnosed via blood smear microscopy or polymerase chain reaction (PCR). If a rapid diagnostic test (RDT) is used to initially diagnose malaria (*28*), the diagnosis must be confirmed either by microscopy or PCR to be counted as a case. Only data from confirmed cases are included in this report.

CDC reviews all reports and might request additional information from the reporting jurisdiction or provider. Rare cases classified as acquired in the United States are investigated further, as are those classified as induced, congenital, or cryptic according to the malaria surveillance definitions. The malaria case report form and instructions for completing it are available from the CDC malaria website (*29*). All data management was done by a CDC malaria surveillance subject matter expert. Data from the structured malaria case report form were entered into the NMSS Microsoft Access database; alternatively, spreadsheets extracted from department of health surveillance systems were normalized and imported into the NMSS Microsoft Access database. For the 2018 analysis, data from electronic case report forms (*30*); the NMSS Microsoft Access database; the CDC Enterprise Laboratory Information Management System; and the NNDSS message validation, processing, and provisioning system (*31*) were imported into a custom Shiny (version 1.7.1; RStudio) data management application, the Malaria Integration Application (MIA). MIA was used for the first time to manage the 2018 record reconciliation and deduplication process. The final data set was exported from MIA to a comma-separated values (.csv) file and imported into SAS (version 9.4; SAS Institute) for cleaning and analysis.

Data elements analyzed include age, sex, pregnancy status, residence, illness onset date, laboratory results, travel history (countries, regions, and dates), chemoprophylaxis (medication used and adherence), history of malaria (date and species), blood transfusion or organ transplant history, hospitalization, clinical complications, treatment medications, illness outcome (survived versus died), and case classification. Data elements with missing values were excluded from analysis.

Pearson’s chi-square test was used to calculate p values and assess differences between variables reported in 2018 compared with previous years. A p value of <0.05 was considered statistically significant. Linear regression using the least squares method was used to assess the linear trend in the number of cases during 1972–2018. The Pearson product-moment correlation coefficient (R^2^) was used to describe the proportion of variation explained by the model. States with one or more cases were categorized into quartiles using the QNTLDEF = 5 option in SAS.

### Definitions

The following definitions are used in malaria surveillance for the United States (*27*) (https://www.cdc.gov/malaria/report.html):

- **Adherence to chemoprophylaxis:** Reported response (yes or no) to the question, “Was chemoprophylaxis taken as prescribed?”
- **Confirmed case:** Symptomatic or asymptomatic infection that occurs in a person in the United States who has laboratory-confirmed (by microscopy or PCR) malaria parasitemia, regardless of whether the person had previous episodes of malaria. A subsequent episode of malaria in the same person is counted as an additional case unless the case is indicated as a treatment failure within 4 weeks of initial presentation.
- **Laboratory criteria for diagnosis:** Demonstration of malaria parasites by blood smear microscopy or PCR.
- **Non-U.S. residents:** Persons who are residents of a country other than the United States. Immigrants and refugees who are establishing residence in the United States are classified as non-U.S. residents if their exposure occurred while they were residents in their originating country.
- **Suspect case:** A positive malaria RDT result in a person in the United States without confirmation by microscopy or PCR.
- **Transfusion transmitted malaria (TTM):** A Plasmodium infection that is accidentally caused by the transfusion of whole blood or blood components from an infected donor to a recipient. In NMSS, the recipient is classified as having induced malaria.
- **Treatment according to CDC recommendations (i.e., appropriate treatment):** Treated with a CDC-recommended regimen appropriate for species, region, and severity of disease (*32*). Patients who received more antimalarial medication than recommended were classified as appropriately treated because the precise sequence and circumstances of excess treatment are not included in the malaria case report and characterizing the purpose or appropriateness of the additional antimalarial treatment is often not possible.
- **U.S. civilians:** U.S. residents, excluding U.S. military personnel.
- **U.S. military personnel:** A person with a case reported by Armed Forces Health Surveillance Division as a current member of the U.S. military, or the reason for travel was a military deployment.
- **U.S. residents:** Persons who live in the United States, including civilian and U.S. military personnel, regardless of legal citizenship. This category does not include recent refugees or immigrants who are establishing residence in the United States if their exposure occurred when they were residents of their originating country.

This report uses terms derived from the World Health Organization (WHO) recommended malaria terminology (*33*) and the CDC *Yellow Book* (*34*). Definitions of the following terms are included for reference:

- **Congenital malaria:** Malaria infection transmitted directly from mother to child during pregnancy or childbirth.
- **Cryptic malaria:** A case of malaria for which epidemiologic investigations cannot identify a plausible mode of acquisition.
- **Imported malaria:** Malaria acquired outside a specific area. In this report, imported cases are those acquired outside the United States.
- **Indigenous malaria:** Local mosquitoborne transmission of malaria with no evidence of importation and no direct link to transmission from an imported case.
- **Induced malaria:** Malaria transmission through a blood transfusion, tissue or organ transplantation, or another parenteral route, not mosquitoborne or congenital transmission.
- **Introduced malaria:** Local mosquitoborne transmission of malaria with strong epidemiological evidence linking the case to an imported case.
- **Radical treatment (or radical cure):** Treatment to kill dormant liver-stage parasites (hypnozoites) of *P. vivax* and *P. ovale* to prevent relapses of malaria.
- **Relapsing malaria:** Recurrence of disease after it has been apparently cured. In malaria, true relapses are caused by activation of dormant liver-stage parasites (hypnozoites) of *P. vivax* and *P. ovale* only. Hypnozoite activation is typically delayed after the primary exposure (*35*); therefore, likely relapses of *P. vivax* and *P. ovale* are defined as occurring >45 days after travel to an area where malaria is endemic.
- **Severe malaria:** A case of malaria with one or more of the following manifestations: neurologic symptoms, acute kidney injury, severe anemia (hemoglobin [Hb] <7g/dL), acute respiratory distress syndrome, or ≥5% parasitemia (*32*,*36*,*37*). Parasitemia can be determined using both thick and thin smears and is calculated using published formulas (https://www.cdc.gov/dpdx/resources/pdf/benchAids/malaria/Parasitemia_and_LifeCycle.pdf). Cases also were counted as severe if the person received treatment for severe malaria (i.e., artesunate, quinidine, or an exchange transfusion) despite having no specific severe manifestations reported. All fatal cases, for which malaria was the cause of death, were classified as severe.
- **Travelers visiting friends and relatives (VFR):** Immigrants, ethnically and racially distinct from the major population of the country of residence (a country where malaria is not endemic), who return to their homeland (a country where malaria is endemic) to visit friends and relatives; family members of immigrants (e.g., spouse or children) are included in this group, even if they were born in the country of residence (*38*,*39*). Non-U.S. residents who travel to visit friends and relatives in the United States also are classified as VFR travelers; however, characteristics of these persons are assessed separately from U.S. resident VFR travelers.

### Diagnosis of Malaria

Three laboratory tests can be used to diagnose malaria: 1) microscopic analysis of a peripheral blood smear, 2) PCR, and 3) RDT; a blood smear is recommended as the preferred first-line test. If malaria is suspected, a Giemsa-stained smear of the patient’s peripheral blood should be examined by microscopy for parasites as soon as possible. Microscopy provides both speciation and an estimation of the level of parasitemia, which is necessary to prescribe appropriate treatment. Diagnostic accuracy depends on blood smear quality and examination by experienced laboratory personnel (*40*,*41*). Three sets of thick and thin blood smears spaced 12–24 hours apart are needed to rule out malaria. PCR, although not timely enough to be useful in the initial diagnosis and treatment of acute malaria, is used to confirm the species, which is important for *P. vivax* and *P. ovale* infections that require additional treatment to prevent relapse (*32*), and for the confirmation of mixed species infections.

The BinaxNow malaria RDT (Abbott Laboratories) detects circulating malaria-specific antigens and is the only RDT approved by the Food and Drug Administration (FDA) for use by hospital and commercial laboratories (*28*); the test is not approved as a point-of-care test by clinicians or the general public (*28*,*42*). RDTs are less sensitive than blood smear microscopy and not able to determine all *Plasmodium* species or quantify malaria parasites; therefore, the results require confirmation and species identification by microscopy (*43*). If microscopy is not performed, although suboptimal because it is not timely for immediate treatment decisions, PCR can confirm an RDT result and determine the species.

### Drug Resistance Marker Surveillance

In 2012, CDC began molecular surveillance of imported malaria cases, with the goal of detecting and characterizing parasites that carry genetic markers (typically single nucleotide polymorphisms in one or more loci or gene copy number variation) associated with antimalarial drug resistance (*44*). Molecular surveillance data are used to identify where drug-resistant foci might be present or emerging in specific parts of the world where malaria is endemic. For each specimen submitted, species confirmation is conducted using a real-time PCR assay capable of detecting the four primary human-infecting *Plasmodium* species. Submitted specimens containing *P. falciparum* parasites are tested for molecular markers associated with resistance to sulfadoxine, pyrimethamine, chloroquine, mefloquine, atovaquone, and artemisinin. Additional markers of resistance in *P. falciparum* or other species will be similarly evaluated as they become available and as new laboratory methods are developed.

Specimens for molecular resistance monitoring are processed by PCR amplification of parasite DNA using appropriate primers for the genes of interest in nested PCR assays as previously described (*45*–*49*) and sequenced by the Sanger method using the ABI 3130 capillary sequencer (Thermo Fisher Scientific). Fragments of genes encoding molecular targets of chloroquine (chloroquine resistance transporter gene, *pfcrt*), pyrimethamine and proguanil (dihydrofolate reductase gene, *pfdhfr*), sulfadoxine (dihydropteroate synthase gene, *pfdhps*), atovaquone (cytochrome b gene, *pfcytb*), mefloquine (multidrug resistance one protein gene, *pfmdr-1*), and artemisinin (kelch 13-propeller domain, *pfk13*) are analyzed for polymorphisms by comparing each sequence to the reference genome. The sequence data are analyzed using Geneious Pro R8 (Biomatters).

**Chloroquine resistance markers:** The *pfcrt* gene sequence is amplified using a nested PCR method as previously described (*45*) and analyzed to identify polymorphisms at codons C72S, M74I, N75E, and K76T.

**Pyrimethamine and proguanil resistance markers:** The *pfdhfr* gene sequence is amplified using a nested PCR method as previously described (*45*) and analyzed to identify polymorphisms at codons A16V, C50R, N51I, C59R, S108T/N, and I164L.

**Sulfadoxine resistance markers:** The *pfdhps* gene sequence is amplified using a nested PCR method as previously described (*45*) and analyzed to identify polymorphisms at codons S436A, A437G, K540E, A581G, and A631S/T.

**Atovaquone resistance markers:** The *pfcytb* gene sequence is amplified using a nested PCR method as previously described (*46*) and analyzed to identify polymorphisms at codons I258M and Y268S.

**Mefloquine resistance markers:** A real-time PCR assay is used to determine the variation in the number of copies of the *pfmdr-1* gene using the comparative cycle threshold (ΔΔC_T_) method as previously described (*47*). DNA from the 3D7 laboratory control, which has a single copy of *pfmdr-1,* is used as the calibrator. In addition, DNA from Indochina W2mef and Dd2 are used as multiple copy number controls.

**Artemisinin resistance markers:** The *pfk13* gene for artemisinin resistance is amplified using a nested PCR method as previously described (*47*–*49*) and analyzed to identify polymorphisms in 10 codons within the propeller domain validated as molecular markers of artemisinin resistance: F446I, N458Y, M476I, Y493H, R539T, I543T, P553L, R561H, P574L, and C580Y (*50*).

## Results

### General Surveillance

**FIGURE 1:**
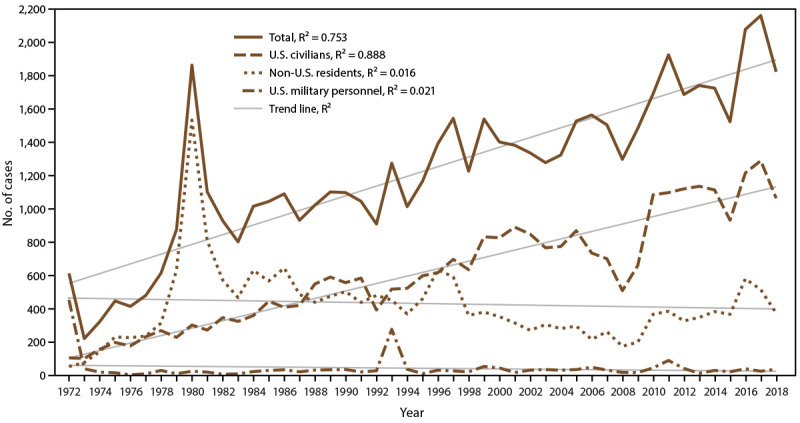
Number of malaria cases* among U.S. civilians, U.S. military personnel, and non-U.S. residents — United States, 1972–2018 **Abbreviation:** R^2^ = square of the Pearson product moment correlation coefficient. * 2018: N = 1,823.

CDC received 1,823 reports of confirmed malaria cases among persons tested in the United States with onset of symptoms in 2018 (Table 2). These cases represented a 15.6% relative decrease from 2017 (n = 2,161), which had the most reported confirmed cases since 1971 when there were 3,180 cases (*51*) (Figure 1). Of the 1,823 cases reported in 2018, a total of 1,788 (98.1%) were imported from countries where malaria was endemic, one case was induced from a bone marrow transplant, and one was investigated and classified as cryptic. Of the 33 (1.8%) cases that were unable to be classified, 15 of these reports contained only laboratory or demographic information, and 18 patients were lost to follow-up for the case investigation. In 2018, of the 1,823 confirmed cases, 58.5% occurred among U.S. civilians, 2.1% among U.S. military personnel, 20.6% among non-U.S. residents, and 18.9% among persons for whom residence status was not reported (Table 2 and Table 3). In 2018, seven persons in the United States died from malaria.

**TABLE 2:** Number of malaria cases* among U.S. military personnel, U.S. civilians, and non-U.S residents — United States, 1970–2018

Year	U.S. military personnel	U.S. civilians	Non-U.S. residents	Status not recorded	Total
	No. (%)	No. (%)	No. (%)	No. (%)	No. (%)
1970	4,096 (96.4)	90 (2.1)	44 (1.0)	17 (0.4)	**4,247 (100)**
1971	2,975 (93.6)	79 (2.5)	69 (2.2)	57 (1.8)	**3,180 (100)**
1972	454 (73.9)	106 (17.3)	54 (8.8)	0 (0.0)	**614 (100)**
1973	41 (18.5)	103 (46.4)	78 (35.1)	0 (0.0)	**222 (100)**
1974	21 (6.5)	158 (48.9)	144 (44.6)	0 (0.0)	**323 (100)**
1975	17 (3.8)	199 (44.4)	232 (51.8)	0 (0.0)	**448 (100)**
1976	5 (1.2)	178 (42.9)	227 (54.7)	5 (1.2)	**415 (100)**
1977	11 (2.3)	233 (48.4)	237 (49.3)	0 (0.0)	**481 (100)**
1978	31 (5.0)	270 (43.8)	315 (51.1)	0 (0.0)	**616 (100)**
1979	11 (1.3)	229 (26.1)	634 (72.3)	3 (0.3)	**877 (100)**
1980	26 (1.4)	303 (16.3)	1,534 (82.3)	1 (0.1)	**1,864 (100)**
1981	21 (1.9)	273 (24.8)	809 (73.3)	0 (0.0)	**1,103 (100)**
1982	8 (0.9)	348 (37.4)	574 (61.7)	0 (0.0)	**930 (100)**
1983	10 (1.2)	325 (40.5)	468 (58.3)	0 (0.0)	**803 (100)**
1984	24 (2.4)	360 (35.4)	632 (62.2)	0 (0.0)	**1,016 (100)**
1985	31 (3.0)	446 (42.7)	568 (54.4)	0 (0.0)	**1,045 (100)**
1986	35 (3.2)	410 (37.6)	646 (59.2)	0 (0.0)	**1,091 (100)**
1987	23 (2.5)	421 (45.2)	488 (52.4)	0 (0.0)	**932 (100)**
1988	33 (3.2)	550 (53.8)	440 (43.0)	0 (0.0)	**1,023 (100)**
1989	35 (3.2)	591 (53.6)	476 (43.2)	0 (0.0)	**1,102 (100)**
1990	36 (3.3)	558 (50.8)	504 (45.9)	0 (0.0)	**1,098 (100)**
1991	22 (2.1)	585 (55.9)	439 (42.0)	0 (0.0)	**1,046 (100)**
1992	29 (3.2)	394 (43.3)	481 (52.9)	6 (0.7)	**910 (100)**
1993	278 (21.8)	519 (40.7)	453 (35.5)	25 (2.0)	**1,275 (100)**
1994	38 (3.7)	524 (51.7)	370 (36.5)	82 (8.1)	**1,014 (100)**
1995	12 (1.0)	599 (51.3)	461 (39.5)	95 (8.1)	**1,167 (100)**
1996	32 (2.3)	618 (44.4)	636 (45.7)	106 (7.6)	**1,392 (100)**
1997	28 (1.8)	698 (45.2)	592 (38.3)	226 (14.6)	**1,544 (100)**
1998	22 (1.8)	636 (51.8)	361 (29.4)	208 (17.0)	**1,227 (100)**
1999	55 (3.6)	833 (54.1)	381 (24.7)	271 (17.6)	**1,540 (100)**
2000	46 (3.3)	827 (59.0)	354 (25.2)	175 (12.5)	**1,402 (100)**
2001	18 (1.3)	891 (64.4)	316 (22.8)	158 (11.4)	**1,383 (100)**
2002	33 (2.5)	849 (63.5)	272 (20.3)	183 (13.7)	**1,337 (100)**
2003	36 (2.8)	767 (60.0)	306 (23.9)	169 (13.2)	**1,278 (100)**
2004	32 (2.4)	775 (58.5)	282 (21.3)	235 (17.7)	**1,324 (100)**
2005	36 (2.4)	870 (56.9)	297 (19.4)	325 (21.3)	**1,528 (100)**
2006	50 (3.2)	736 (47.1)	217 (13.9)	561 (35.9)	**1,564 (100)**
2007	33 (2.2)	701 (46.6)	263 (17.5)	508 (33.8)	**1,505 (100)**
2008	19 (1.5)	510 (39.3)	176 (13.6)	593 (45.7)	**1,298 (100)**
2009	18 (1.2)	661 (44.5)	201 (13.5)	604 (40.7)	**1,484 (100)**
2010	46 (2.7)	1,085 (64.2)	368 (21.8)	192 (11.4)	**1,691 (100)**
2011	91 (4.7)	1,098 (57.0)	386 (20.1)	350 (18.2)	**1,925 (100)**
2012	43 (2.5)	1,121 (66.4)	328 (19.4)	195 (11.6)	**1,687 (100)**
2013	14 (0.8)	1,136 (65.2)	349 (20.0)	242 (13.9)	**1,741 (100)**
2014	31 (1.8)	1,114 (64.6)	384 (22.3)	196 (11.4)	**1,725 (100)**
2015	23 (1.5)	933 (61.2)	368 (24.2)	200 (13.1)	**1,524 (100)**
2016	41 (2.0)	1,216 (58.5)	581 (28.0)	240 (11.5)	**2,078 (100)**
2017	26 (1.2)	1,290 (59.7)	516 (23.9)	329 (15.2)	**2,161 (100)**
2018	38 (2.1)^†^	1,066 (58.5)	375 (20.6)^†^	344 (18.9)^†^	**1,823 (100)**

* A case was defined as symptomatic or asymptomatic illness that occurs in the United States or one of its territories in a person who has laboratory-confirmed malaria parasitemia (microscopy or polymerase chain reaction), regardless of whether the person had previous episodes of malaria. A subsequent episode of malaria occurring in a person is counted as an additional case unless it occurred as a result of a drug resistance failure. Relapsing illnesses are counted as a subsequent case.

† Denotes 2018 values that are statistically different (Pearson’s chi-square, p<0.5) from those in 2017.

**TABLE 3:** Number of malaria cases by demographics, region of acquisition, and primary reason for travel, by subject resident status — United States, 2018

Characteristic	U.S. military personnel			U.S. civilians			Non-U.S. residents			Status not recorded			Total		
	No.	(%)*	(%)^†^	No.	(%)*	(%)^†^	No.	(%)*	(%)^†^	No.	(%)*	(%)^†^	No.	(%)*	(%)**^†^**
**Total**	**38**	**(2.1)**	**—^§^**	**1,066**	**(58.5)**	**—^§^**	**375**	**(20.6)**	**—^§^**	**344**	**(18.9)**	**—^§^**	**1,826**	**(100)**	**—^§^**
**Sex**															
Male	36	(94.7)	—^¶^	671	(63.0)	—^¶^	214	(57.1)	(57.2)	205	(59.6)	(61.9)	**1,126**	**(61.8)**	**(62.2)**
Female	2	(5.3)	—^¶^	395	(37.1)	—^¶^	160	(42.7)	(42.8)	126	(36.6)	(38.1)	**638**	**(37.5)**	**(37.8)**
Unknown	0	(0.0)	—^¶,^**	0	(0.0)	—^¶,^**	1	(0.3)	—**	13	(3.8)	—**	**14**	**(0.8)**	—**
**Age (yrs)**															
<18	0	(0.0)	—^††^	131	(12.3)	—^††^	130	(34.7)	—^††^	33	(9.6)	—^††^	**294**	**(16.1)**	**—^††^**
18–64	38	(100)	—^††^	840	(78.8)	—^††^	225	(60.0)	—^††^	294	(85.5)	—^††^	**1,397**	**(76.6)**	**—^††^**
≥65	0	(0.0)	—^††^	95	(8.9)	—^††^	20	(5.3)	—^††^	17	(4.9)	—^††^	**132**	**(7.2)**	**—^††^**
**Race and ethnicity**															
Ethnicity															
Not Hispanic or Latino	16	(42.1)	(76.2)	835	(78.3)	(97.3)	296	(78.9)	(97.4)	209	(60.8)	(100.0)	**1,356**	**(74.4)**	**(97.4)**
Hispanic or Latino	5	(13.2)	(23.8)	23	(2.2)	(2.7)	8	(2.1)	(2.6)	0	(0.0)	(0.0)	**36**	**(2.0)**	**(2.6)**
Unknown	17	(44.7)	—**	2.8	(19.5)	—**	71	(18.9)	—**	135	(39.2)	—**	**431**	**(23.6)**	—**
Race															
Asian	0	(0.0)	(0.0)	31	(2.9)	(3.2)	39	(10.4)	(11.5)	41	(11.9)	(15.7)	**111**	**(6.1)**	**(3.9)**
Black or African American	11	(29.0)	(33.3)	769	(72.2)	(78.8)	265	(70.7)	(78.4)	179	(51.9)	(68.3)	**1,224**	**(67.1)**	**(76.1)**
White	20	(56.2)	(60.6)	134	(12.6)	(13.7)	17	(4.5)	(5.0)	33	(9.6)	(12.6)	**204**	**(11.2)**	**(12.7)**
Other^§§^	2	(5.3)	(6.1)	42	(3.9)	(4.3)	17	(4.5)	(5.0)	9	(2.6)	(3.4)	**70**	**(3.8)**	**(4.4)**
Unknown	5	(18.4)	—**	90	(12.3)	—**	37	(14.4)	—**	82	(26.7)	—**	**214**	**(15.6)**	—**
**Region of acquisition^¶¶^**															
Africa	12	(31.6)	(32.4)	970	(91.3)	(92.3)	304	(81.1)	(81.7)	233	(81.1)	(88.9)	**1,519**	**(85.0**	**(88.2)**
West Africa, unspecified	5	(13.5)	(13.5)	713	(67.0)	(67.8)	170	(45.3)	(45.7)	173	(55.6)	(66.0)	**1,061**	**(59.3)**	**(61.6)**
Asia	25	(65.8)	(67.6)	41	(3.9)	(3.9)	54	(14.4)	(14.5)	21	(6.8)	(8.0)	**141**	**(7.9)**	**(8.2)**
South America	0	(0.0)	(0.0)	16	(1.5)	(1.5)	12	(3.2)	(3.2)	7	(2.7)	(2.7)	**35**	**(2.0)**	**(2.0)**
Central America or the Caribbean	0	(0.0)	(0.0)	19	(1.8)	(1.8)	2	(0.5)	(0.5)	0	(0.0)	(0.0)	**21**	**(1.2)**	**(1.2)**
Oceania	0	(0.0)	(0.0)	5	(0.5)	(0.5)	0	(0.0)	(0.0)	1	(0.3)	(0.4)	**6**	**(0.3)**	**(0.4)**
Unknown	1	(2.3)	—**	13	(1.2)	—**	3	(0.8)	—**	49	(15.7)	—**	**66**	**(3.7)**	—**
Total	38	(100)	(100)	1,064	(100)	(100)	375	(100)	(100)	311	(100)	(100)	**1,788**	**(100)**	**(100)**
**Primary reason for travel^¶¶^**															
Visiting friends and relatives	4	(10.5)	(10.5)	673	(63.3)	(77)	82	(21.9)	(26.0)	74	(23.7)	(71.8)	**833**	**(46.6)**	**(62.6)**
Tourist	0	(0)	(0)	71	(6.7)	(8.1)	6	(1.6)	(1.9)	9	(2.9)	(8.7)	**86**	**(4.8)**	**(6.5)**
Missionary or dependent	0	(0)	(0)	45	(4.2)	(5.2)	5	(1.3)	(1.6)	5	(1.6)	(4.9)	**55**	**(3.1)**	**(4.1)**
Business	0	(0)	(0)	61	(5.7)	(7.0)	14	(3.7)	(4.4)	6	(1.9)	(5.8)	**81**	**(4.5)**	**(6.1)**
Student or teacher	0	(0)	(0)	20	(1.9)	(2.3)	15	(4.0)	(4.8)	7	(2.2)	(6.8	**42**	**(2.4)**	**(3.1)**
Air crew or sailor	0	(0)	(0)	1	(0.1)	(0.1)	3	(0.8)	(1.0)	0	(0)	(0)	**4**	**(0.2)**	**(0.3)**
Peace Corps	0	(0)	(0)	1	(0.1)	(0.1)	0	(0)	(0)	0	(0)	(0)	**1**	**(0.1)**	**(0.1)**
Refugee or immigrant	0	(0)	(0)	0	(0)	(0)	183	(40.8)	(58.1)	0	(0)	(0)	**183**	**(10.2)**	**(13.8)**
Military deployment	34	(89.5)	(89.5)	0	(0)	(0)	2	(0.5)	(0.6)	0	(0)	(0)	**36**	**(2.0)**	**(2.7)**
Other	0	(0)	(0)	2	(0.2)	(0.2)	5	(1.3)	(1.6)	2	(0.6)	(1.9)	**9**	**(0.5)**	**(0.7)**
Unknown	0	(0)	—**	190	(19.9	—**	60	(16)	—**	208	(66.7)	—**	**458**	**(25.6)**	—**
Total	38	(100)	(100)	1,064	(100)	(100)	375	(100)	(100)	311	(100)	(100)	**1,788**	**(100)**	**(100)**

* Percentage calculated among all subjects.

^†^ Percentage calculated among subjects with known responses.

^§^ Not applicable because all cases were categorized by resident status.

^¶^ Not applicable because sex was known for all subjects.

** Not applicable because unknown responses were excluded from the calculation.

^††^ Not applicable because age was known for all subjects.

^§§^ Other race includes American Indian or Alaska Native, Native Hawaiian or Pacific Islander, mixed race, or another race not specified.

^¶¶^ Among imported cases.

Since 1972, the numbers of malaria cases have been increasing, with an average gain of 29.2 cases per year (R^2^ = 0.753) (Table 2 and Figure 1). The number of malaria cases among the U.S. civilian population has increased over time, with approximately 22 cases added each year during 1972–2018 (R^2^ = 0.888). In contrast, the trajectory of the accumulation of military and non-U.S. resident cases was flat during this period (R^2^ = 0.021 and R^2^ = 0.016, respectively) (Figure 1).

Among all cases identified in the United States, a total of 61.8% of patients were male, 76.6% were adults aged 18–64 years, and 67.1% were Black or African American (76.1% among patients with known race), and 2.0% were Hispanic or Latino (Table 3). A total of 294 (16.1%) infants, children, and adolescents aged <18 years received a diagnosis of malaria in the United States in 2018, and 66 (22.5%) of these were aged <5 years. In 2018, a total of 132 persons with malaria were aged ≥65 years.

The primary reason for travel was reported for 1,330 (74.4%) of 1,788 imported cases (Table 3). Of these, more than one half of all cases (833 [62.6%]) and more than three fourths of U.S. civilian cases (673 [77.0%]) reported traveling to visit friends and relatives (VFR). In 2018, the number and proportion of cases in non-U.S. residents that traveled to the United Sates to visit family or friends decreased (82 cases [26.0%] in 2018 compared with 144 cases [35.2%] in 2017). Traveling to the United States as an immigrant or refugee was reported for 183 patients (13.8%). Others reported traveling for tourism (86 cases [6.5%]), business (81 cases [6.1%]), missionary (55 cases [4.1%]), or for education as a student or teacher (42 cases [3.1%]). Traveling as part of an air or ship crew, Peace Corps, foreign military, or for other reasons was reported by <1.0% of patients. In 2018, a total of 183 (58.1%) non-U.S. residents with malaria reported having traveled to the United States as a refugee or immigrant. This is a proportional increase compared with the 186 cases (45.5%) in non-U.S. residents who had traveled to the United States as a refugee or immigrant in 2017.

**FIGURE 2:**
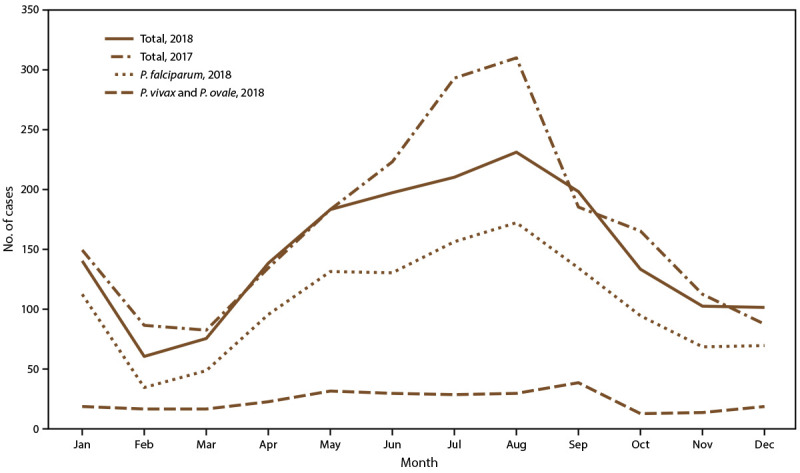
Number* of imported malaria cases, by *Plasmodium* species and month of symptom onset — United States, 2017^†^ and 2018^§^ * 2017: N = 2,009; 2018: N = 1,768. ^†^ Information about the month of illness onset was available for 2,009 (95.1%) of 2,112 imported cases. ^§^ Information about the month of illness onset was available for 1,768 (98.9%) of 1,788 imported cases. The mean number of imported cases per month in 2018 was 147.3.

Among the 1,788 cases of malaria imported into the United States in 2018, a total of 1,768 cases (98.9%) had information reported for the month of illness onset (Figure 2). Overall, the mean number of imported cases per month was 147.3. More cases than average were reported for May–September; the highest number of cases (231) was reported in August followed by July (210) and September (198). The lowest number of cases occurred in February (60 cases) and March (75 cases). *P. falciparum* cases accounted for approximately 70.3% of cases reported with seasonal information, and approximately 103.6 *P. falciparum* cases were reported per month. *P. vivax* and *P. ovale* cases, including 13 mixed infections containing *P. vivax* or *P. ovale*, together accounted for approximately 15.2% of all cases with seasonal information, with an average of 22.4 cases per month. In 2018, the number of imported malaria cases was 324 less than the imported malaria cases in 2017 (2,112 imported malaria cases in 2017, of which 2,009 [95.1%] had information on the month of illness onset). The seasonal trends were similar between the years.

### *Plasmodium* Species

In 2018, a total of 1,607 (88.2%) cases reported the *Plasmodium* species, a similar proportion to 2017 (1,935 cases [89.5%]) (Table 4). Specimens from 209 (11.5%) malaria patients were sent to CDC for confirmatory diagnostic testing and CDC was able to determine the species for 195 (93.3%) of them.

**TABLE 4:** Number of malaria cases, by *Plasmodium* species and year — United States, 2014–2018

Species	2014			2015			2016			2017			2018		
	No.	(%)*	(%)^†^	No.	(%)*	(%)^†^	No.	(%)*	(%)^†^	No.	(%)*	(%)^†^	No.	(%)*	(%)^†^
*P. falciparum*	1,141	(66.1)	(74.9)	1,025	(67.3)	(77.2)	1,419	(68.3)	(76.6)	1,523	(70.5)	(78.7)	1,273	(69.8)	(79.2)
*P. vivax*	230	(13.3)	(15.1)	180	(11.8)	(13.6)	251	(12.1)	(13.6)	216	(10)	(11.2)	173	(9.5)	(10.8)
*P. ovale*	90	(5.2)	(5.9)	63	(4.1)	(4.7)	99	(4.8)	(5.3)	119	(5.5)	(6.2)	95	(5.2)	(5.9)
*P. malariae*	47	(2.7)	(3.1)	48	(3.2)	(3.6)	61	(2.9)	(3.3)	55	(2.6)	(2.8)	48	(2.6)	(3)
*P. knowlesi*	0	(0)	(0)	0	(0)	(0)	0	(0)	(0)	0	(0)	(0)	1	(0.1)	(0.1)
Mixed	15	(0.9)	(1)	12	(0.8)	(0.9)	23	(1.1)	(1.2)	22	(1)	(1.1)	17	(0.9)	(1.1)
Undetermined	202	(11.7)	—^§^	196	(12.9)	—	225	(10.8)	—	226	(10.5)	—	216	(11.9)	—
**Total**	**1,725**	**(100)**	**(100)**	**1,524**	**(100)**	**(100)**	**2,078**	**(100)**	**(100)**	**2,161**	**(100)**	**(100)**	**1,823**	**(100)**	**(100)**

* Percentage among all infections.

^†^ Percentage among infections with known species.

^§^ Not applicable because undetermined species were excluded from the calculation.

Among the 1,607 cases with *Plasmodium* species determined, most cases speciated were *P. falciparum* (1,273 cases) (Table 4). Although this is the highest proportion (79.2%) of *P. falciparum* cases reported during 2014–2018, it is fewer than the numbers of cases identified in 2016 and 2017 (1,419 [76.6%] in 2016 and 1,523 [78.7%] in 2017). During 2014–2018, there was an average increase of 76.2 *P. falciparum* cases per year. In 2018, a total of 173 (10.8%) *P. vivax* cases were confirmed, a similar proportion to what was observed in 2017 (216 cases [11.2%]). During 2014–2018, there has been a decreasing trend in the number of *P. vivax* cases, with an average of 7.8 fewer cases per year. Approximately 6% of cases in 2018 (95 [5.9%] and 2017 (119 [6.2%]) were *P. ovale*; approximately 3% of cases were *P. malariae* (48 [3.0%] in 2018 and 55 [2.8%] in 2017). For the first time since 2008, there was one imported *P. knowlesi* case (*52*,*53*). The adult patient had traveled to Southeast Asia and reported mosquito exposures while in forests. It is unknown if the patient took chemoprophylaxis to prevent malaria. The patient experienced uncomplicated malaria, was treated with atovaquone-proguanil, and recovered.

In 2018, a total of 17 (1.1%) malaria cases with mixed species were reported, a similar proportion to that in 2017 (22 cases [1.1%]). Of the mixed species malaria cases, 14 were from Africa, two from South America, and one from an unknown region. Nine malaria cases with mixed infections were *P. falciparum* and *P. ovale* (seven were PCR confirmed; eight cases came from Africa and one from an unknown country); four cases were *P. falciparum* and *P. malariae* (all PCR confirmed; all came from Africa); two cases were *P. falciparum* and *P. vivax* (both PCR confirmed; both came from South America); one case was *P. malariae* and *P. ovale* (not PCR confirmed; came from Africa); and one was *P. vivax* and *P. ovale* (PCR confirmed; came from Africa).

Of the 1,788 imported malaria cases in 2018, a total of 692 (38.7%) were PCR confirmed (Table 5). Among all 1,823 cases, 705 (38.7%) were PCR confirmed in 2018, more than the proportion of PCR confirmed in 2017 (764 cases [35.4%]). This includes the *P. knowlesi* case, and 82.4% of the reported cases with mixed infections (14 of 17 cases). Approximately one half of all *P. ovale* and *P. malariae* cases were PCR confirmed (51 of 95 [53.7%] of *P. ovale*, and 24 of 48 [50.0%] of *P. malariae*). A total of 542 (42.6%) of *P. falciparum* and 62 (35.8%) of *P. vivax* cases were PCR confirmed. Eleven cases (5.0%) with unknown species reported were PCR confirmed to the *Plasmodium* genus level.

**TABLE 5:** Number of imported malaria cases (including polymerase chain reaction-confirmed cases), by country of acquisition and *Plasmodium* species — United States, 2018

Country of acquisition	*P. falciparum*		*P. vivax*		*P. ovale*		*P. malariae*		*P. knowlesi*		Mixed		Unknown		Total	
	PCR	Total	PCR	Total	PCR	Total	PCR	Total	PCR	Total	PCR	Total	PCR	Total	PCR	Total
**Africa**	**518**	**1,217**	**8**	**31**	**46**	**86**	**23**	**43**	**0**	**0**	**11**	**14**	**11**	**128**	**617**	**1,519**
Angola	2	7	0	0	0	1	0	0	0	0	0	0	0	1	**2**	**9**
Benin	1	4	0	0	0	0	0	0	0	0	0	0	0	0	**1**	**4**
Burkina Faso	8	13	0	0	0	0	0	0	0	0	0	0	0	1	**8**	**14**
Burundi	3	4	0	0	0	0	0	0	0	0	0	0	0	0	**3**	**4**
Cameroon	26	80	0	3	3	3	0	5	0	0	3	3	0	9	**32**	**103**
Central African Republic	0	2	0	0	0	0	0	0	0	0	0	0	0	1	**0**	**3**
Chad	4	7	1	1	0	0	0	1	0	0	0	0	0	0	**5**	**9**
Congo, Democratic Republic of	11	24	0	1	2	3	2	2	0	0	0	0	2	7	**17**	**37**
Equatorial Guinea	1	6	0	0	0	0	0	0	0	0	0	0	0	1	**1**	**7**
Ethiopia	3	6	3	11	0	0	0	0	0	0	0	0	0	1	**6**	**18**
Gabon	2	7	0	0	0	0	1	1	0	0	0	0	0	0	**3**	**8**
Gambia, The	0	1	0	0	0	0	0	0	0	0	0	0	0	0	**0**	**1**
Ghana	63	114	1	3	3	5	0	0	0	0	0	0	1	10	**68**	**132**
Guinea	37	54	0	0	2	2	1	1	0	0	0	0	0	0	**40**	**57**
Guinea-Bissau	1	2	0	0	0	0	0	0	0	0	0	0	0	0	**1**	**2**
Ivory Coast	38	64	0	0	1	3	1	1	0	0	1	1	0	9	**41**	**78**
Kenya	20	51	0	0	0	0	2	3	0	0	0	0	1	3	**23**	**57**
Liberia	39	104	0	0	0	3	1	2	0	0	0	0	1	13	**41**	**122**
Malawi	0	2	0	0	0	0	1	1	0	0	0	0	0	0	**1**	**3**
Mali	7	13	0	0	1	1	0	0	0	0	0	0	0	1	**8**	**15**
Mozambique	0	2	0	0	0	0	1	1	0	0	0	0	0	1	**1**	**4**
Niger	1	6	0	0	0	0	0	0	0	0	0	0	0	0	**1**	**6**
Nigeria	137	316	1	3	18	36	5	8	0	0	2	2	4	37	**167**	**402**
Rwanda	2	4	1	1	0	3	0	0	0	0	0	0	0	0	**3**	**8**
Senegal	2	3	0	0	0	0	1	1	0	0	0	0	0	0	**3**	**4**
Sierra Leone	36	130	0	1	2	3	0	2	0	0	1	2	0	14	**39**	**152**
South Africa	1	3	0	0	0	0	0	0	0	0	0	0	0	0	**1**	**3**
South Sudan	2	10	0	1	1	1	0	0	0	0	0	0	0	0	**3**	**12**
Sudan	9	28	0	1	1	1	0	0	0	0	0	0	0	2	**10**	**32**
Tanzania	8	19	0	0	6	8	1	2	0	0	3	3	1	3	**19**	**35**
Togo	19	38	0	0	0	0	0	0	0	0	1	1	0	1	**20**	**40**
Uganda	11	27	1	2	3	5	3	6	0	0	0	1	0	2	**18**	**43**
Zambia	2	4	0	0	0	0	0	0	0	0	0	0	0	1	**2**	**5**
Zimbabwe	1	1	0	0	0	0	0	0	0	0	0	0	0	0	**1**	**1**
Africa, unspecified	6	27	0	0	2	3	1	2	0	0	0	1	0	2	**9**	**35**
Central Africa, unspecified	0	0	0	0	0	1	0	0	0	0	0	0	0	0	**0**	**1**
East Africa, unspecified	3	8	0	3	1	2	1	3	0	0	0	0	0	3	**5**	**19**
South Africa, unspecified	0	1	0	0	0	0	0	0	0	0	0	0	0	1	**0**	**2**
West Africa, unspecified	12	25	0	0	0	2	1	1	0	0	0	0	1	4	**14**	**32**
**Asia**	**4**	**12**	**40**	**103**	**1**	**4**	**1**	**4**	**1**	**1**	**0**	**0**	**0**	**17**	**47**	**141**
Afghanistan	0	2	18	43	0	2	0	1	0	0	0	0	0	5	**18**	**53**
Cambodia	0	0	0	0	0	0	0	0	0	0	0	0	0	1	**0**	**1**
India	2	6	15	36	0	0	0	1	0	0	0	0	0	10	**17**	**53**
Indonesia	1	2	1	1	0	0	0	0	0	0	0	0	0	0	**2**	**3**
Laos	0	0	0	0	0	0	1	1	0	0	0	0	0	0	**1**	**1**
Pakistan	0	0	5	13	0	1	0	1	0	0	0	0	0	1	**5**	**16**
Philippines	0	0	0	0	1	1	0	0	1	1	0	0	0	0	**2**	**2**
South Korea	0	0	0	6	0	0	0	0	0	0	0	0	0	0	**0**	**6**
Thailand	1	1	1	3	0	0	0	0	0	0	0	0	0	0	**2**	**4**
Vietnam	0	1	0	0	0	0	0	0	0	0	0	0	0	0	**0**	**1**
Southeast Asia, unspecified	0	0	0	1	0	0	0	0	0	0	0	0	0	0	**0**	**1**
**Central America and the Caribbean**	**5**	**8**	**0**	**10**	**0**	**0**	**0**	**0**	**0**	**0**	**0**	**0**	**0**	**3**	**5**	**21**
Dominican Republic	2	3	0	0	0	0	0	0	0	0	0	0	0	0	**2**	**3**
Guatemala	0	0	0	0	0	0	0	0	0	0	0	0	0	1	**0**	**1**
Haiti	3	5	0	0	0	0	0	0	0	0	0	0	0	1	**3**	**6**
Honduras	0	0	0	1	0	0	0	0	0	0	0	0	0	0	**0**	**1**
Nicaragua	0	0	0	6	0	0	0	0	0	0	0	0	0	1	**0**	**7**
Panama	0	0	0	1	0	0	0	0	0	0	0	0	0	0	**0**	**1**
Central America, unspecified	0	0	0	2	0	0	0	0	0	0	0	0	0	0	**0**	**2**
**South America**	**0**	**2**	**11**	**23**	**0**	**0**	**0**	**0**	**0**	**0**	**2**	**2**	**0**	**8**	**13**	**35**
Brazil	0	0	1	3	0	0	0	0	0	0	0	0	0	0	**1**	**3**
Colombia	0	0	0	0	0	0	0	0	0	0	0	0	0	1	**0**	**1**
Guyana	0	0	7	9	0	0	0	0	0	0	2	2	0	0	**9**	**11**
Peru	0	2	1	4	0	0	0	0	0	0	0	0	0	6	**1**	**12**
Venezuela	0	0	2	6	0	0	0	0	0	0	0	0	0	0	**2**	**6**
South America, unspecified	0	0	0	1	0	0	0	0	0	0	0	0	0	1	**0**	**2**
**Oceania**	**3**	**3**	**2**	**2**	**0**	**0**	**0**	**1**	**0**	**0**	**0**	**0**	**0**	**0**	**5**	**6**
Papua New Guinea	3	3	2	2	0	0	0	1	0	0	0	0	0	0	**5**	**6**
**Unknown**	**2**	**13**	**1**	**3**	**1**	**1**	**0**	**0**	**0**	**0**	**1**	**1**	**0**	**48**	**5**	**66**
**Total**	**532**	**1,255**	**62**	**172**	**48**	**91**	**24**	**48**	**1**	**1**	**14**	**17**	**11**	**204**	**692**	**1,788**

**Abbreviation:** PCR = polymerase chain reaction.

Among the 1,584 imported malaria cases with the *Plasmodium* species determined, 1,238 (78.2%) had complete information on the travel return and illness onset dates allowing for calculation of the interval between these dates (Table 6). Among these patients, regardless of infecting species, 173 (14.0%) had illness onset before the return to the United States and 1,063 (85.9%) had illness onset before or within 29 days of arrival to the United States. Among patients with *P. falciparum*, 99% had illness onset before return to, or less than 90 days from the date of arrival to the United States (994 of 1,004 cases). In contrast, approximately 40% of *P. vivax* and *P. ovale* patients had illness onset 90 or more days after their return to the United States (51 [40.8%] of *P. vivax* cases, and 26 [40.6%] of *P. ovale* cases), consistent with the potential for these species to relapse because of the persistence of liver hypnozoites, or to have an extended incubation period before symptom onset (*4*,*6*–*8*). Of infections with any species reported, 98.9% (1,224 cases) had illness onset within 1 year of returning from a country where malaria is endemic. Twelve of 14 infections that occurred more than 1 year after return to the United States were *P. vivax* (eight cases) and *P. ovale* (four cases). Of the two *P. falciparum* cases with illness reported more than 1 year after return, one was a PCR-confirmed infection in an asymptomatic bone marrow transplant donor (see induced case summary for more information), and the other case was blood smear confirmed infection in a U.S. resident who had traveled to West Africa,^§^ with no reason for travel or travel duration reported.

**TABLE 6:** Number and percentage of imported malaria cases, by *Plasmodium* species* and interval between date of arrival in the United States and onset of illness — United States, 2018

Interval (days)	*P. falciparum* No. (%)	*P. vivax* No. (%)	*P. ovale* No. (%)	*P. malariae* No. (%)	Mixed No. (%)	Total No. (%)
<0^†^	153 (15.2)	11 (8.8)	6 (9.4)	2 (6.1)	1 (8.3)	**173 (14.0)**
0–29	799 (79.6)	41 (32.8)	22 (34.4)	20 (60.6)	8 (66.7)	**890 (71.9)**
30–89	42 (4.2)	22 (17.6)	10 (15.6)	7 (21.2)	2 (16.7)	**83 (6.7)**
90–179	5 (0.5)	17 (13.6)	7 (10.9)	3 (9.1)	1 (8.3)	**33 (2.7)**
180–364	3 (0.3)	26 (20.8)	15 (23.4)	1 (3.0)	0 (0)	**45 (3.6)**
≥365	2 (0.2)	8 (6.4)	4 (6.3)	0 (0)	0 (0)	**14 (1.1)**
**Total**	**1,004 (100.0)**	**125 (100.0)**	**64 (100.0)**	**33 (100.0)**	**12 (100.0)**	**1,238 (100.0)**

* This table does not include one case of *P. knowlesi* because of incomplete travel dates. Persons for whom *Plasmodium* species, date of arrival in the United States, or date of onset of illness is unknown are not included.

^†^ Cases in this row are among patients who had onset of illness before arriving in the United States.

Among all 1,823 malaria cases, information on history of malaria for the infected patient in the previous year was available for 1,285 (70.5%) cases; of these, 244 (19.0%) patients had malaria previously, and all cases were imported. Of the 14 mixed species infections with information, eight (57.1%) patients reported a previous history of malaria, as did 48.0% (61 of 127 cases) of patients with *P. vivax*, 27.8% (20 of 72 cases) with *P. ovale*, 22.2% (eight of 36 cases) with *P. malariae*, and 13.8% (128 of 927 cases) with *P. falciparum* infections. Among cases where the species of the current illness was known, the species of the previous infection was identified for 43 patients with a history of malaria (22 were *P. falciparum*, 19 were *P. vivax*, and one each was *P. ovale* and *P. malariae*). Concordance between the species of the acute illness and the reported prior illness was observed for 37 (86.1%) of 43 reports containing information on the species of the prior infection (19 were *P. vivax* infections, 17 were *P. falciparum*, and one was *P. ovale*). The 20 cases in patients currently and previously infected with *P. vivax* or *P. ovale* were considered possible relapses because these species can retain dormant hypnozoites, and the previously reported infection was with the same species; other relapse cases might have occurred, although they could not be classified as such because of missing information reported. Fourteen (70.0%) possible relapse cases had information on the time between the previous and current illnesses; the mean number of days between previous and relapsing illness was 187.6 days (range: 52–377 days). Of the 19 likely *P. vivax* relapse cases, eight originated from Africa, six cases from Asia, three cases were from South America, and one each from Oceania and Central America. One likely *P. ovale* relapse case was from Africa. Date information for the previous illness was reported for six of 17 *P. falciparum* infections with a history of malaria and concordant prior species reported, and three (50.0%) of these indicated that the prior illness occurred within 35 days from the current illness, suggesting that treatment failure might have contributed to the current illness diagnosed in the United States.

### Region of Acquisition and Diagnosis

The region of travel was reported for 1,722 (96.3%) of the 1,788 imported cases (Table 3). Of these, 1,519 (88.2%) cases were acquired in Africa, 141 (8.2%) in Asia, 35 (2.0%) in South America, 21 (1.2%) in Central America and the Caribbean, and six (<1.0%) in Oceania. Of the 1,519 cases acquired in Africa, 1,061 cases (69.8%) were from West Africa. No differences in the proportions of cases by region in 2018 were observed compared with 2017; however, in 2018, there was an increase in cases with an unknown region of acquisition (66 cases [3.7%] in 2018 versus 39 cases [1.9%] in 2017). The proportion of cases from Africa was comparable in 2018 (88.2%) and 2017 (87.8%), and in both years, approximately two thirds of cases acquired in Africa originated from West Africa (1,061 of 1,519 cases [69.8%] in 2018; 1,216 of 1,819 cases [66.9%] in 2017). Four of the five countries where the highest number of cases were acquired in 2018 were within West Africa (Nigeria, 402 cases; Sierra Leone, 152 cases; Ghana, 132 cases; Liberia, 122 cases); 103 cases were acquired from Cameroon, in Central Africa, which is the fifth highest country of acquisition. The top five countries of acquisition were the same in 2017. Ninety-two percent of U.S. civilians acquired malaria in Africa, (970 [92.3%]), and among these, 713 (73.5%) were from West Africa; this is a higher proportion than in 2017 (1,162 [90.1%] cases from Africa, and of these, 796 [68.5%] cases from West Africa). In 2018, a greater proportion of U.S. civilians (970 cases [92.3%]) had acquired malaria from Africa than non-U.S. resident (304 cases [81.7%]). Likewise, a greater proportion of U.S. residents acquired malaria from West Africa (713 [67.8%]) compared with non-U.S. residents (170 [45.7%]).

A similar proportion of imported malaria cases was acquired from Asia in 2018 (7.9%) as in 2017 (8.6%), although there were 40 fewer cases from this region in 2018 (181 cases in 2017 versus 141 cases in 2018). Most of the cases (122 cases [86.5%]) acquired from Asia in 2018 originated from India (53 cases), Afghanistan (53 cases), and Pakistan (16 cases). A decrease in cases from India accounts for the fewer cases originating from Asia (105 cases from India in 2017 versus 53 cases from India in 2018); whereas the numbers of cases from Afghanistan and Pakistan were steady in these years (48 and 53 cases from Afghanistan, and 18 and 16 cases from Pakistan, in 2017 and 2018, respectively). Less than 4% of U.S. residents acquired malaria from Asia (40 cases [3.9%]), compared with 14.5% of non-U.S. residents (54 cases).

Less than 2% of malaria cases were acquired from South America (35 cases [2.0%]) and from Central America or the Caribbean (21 cases [1.2%]), comparable to cases from these regions in 2017. As in 2017, in 2018, six (<1.0%) persons acquired malaria from Papua New Guinea in the Oceania region.

The primary reason for travel and the region of acquisition were known for 1,321 (73.9%) of 1,788 imported cases (Table 7). Of 828 patients who were VFR travelers, 776 (93.7%) traveled in Africa, and VFR traveling accounted for 66.7% of cases acquired in Africa. A total of 143 refugee or immigrant patients (79.0%) traveled from Africa, 37 (20.4%) from Asia, and one from Central America and the Caribbean (<1%).

**TABLE 7:** Number and percentage of imported cases among U.S. military personnel, U.S. civilians, and non-U.S residents, by purpose of travel at the time of malaria acquisition – United States, 2018

Category	Region					
	Africa No. (%)	Asia No. (%)	South America No. (%)	Central America and the Caribbean No. (%)	Oceania No. (%)	Total No. (%)
Visiting friends and relatives	776 (66.7)	36 (30.8)	7 (31.8)	9 (56.3)	0 (0)	**828 (62.7)**
Tourist	66 (5.7)	10 (8.9)	7 (31.8)	3 (18.8)	0 (0)	**86 (6.5)**
Missionary or dependent	48 (4.1)	1 (0.9)	2 (9.1)	2 (12.5)	1 (33.3)	**54 (4.1)**
Business	70 (6)	3 (2.6)	5 (22.7)	1 (6.3)	2 (66.7)	**81 (6.1)**
Student or teacher	41 (3.5)	0 (0)	1 (4.6)	0 (0)	0 (0)	**42 (3.2)**
Air crew or sailor	1 (0.1)	3 (2.6)	0 (0)	0 (0)	0 (0)	**4 (0.3)**
Peace Corps	0 (0)	1 (0.9)	0 (0)	0 (0)	0 (0)	**1 (0.1)**
Refugee or immigrant	143 (12.3)	37 (31.6)	0 (0)	1 (6.3)	0 (0)	**181 (13.7)**
Military deployment	9 (0.8)	26 (22.2)	0 (0)	0 (0)	0 (0)	**35 (2.7)**
Other	9 (0.8)	0 (0)	0 (0)	0 (0)	0 (0)	**9 (0.7)**
**Total**	**1,163 (100)**	**117 (100)**	**22 (100)**	**16 (100)**	**3 (100)**	**1,321 (100)**

**FIGURE 3:**
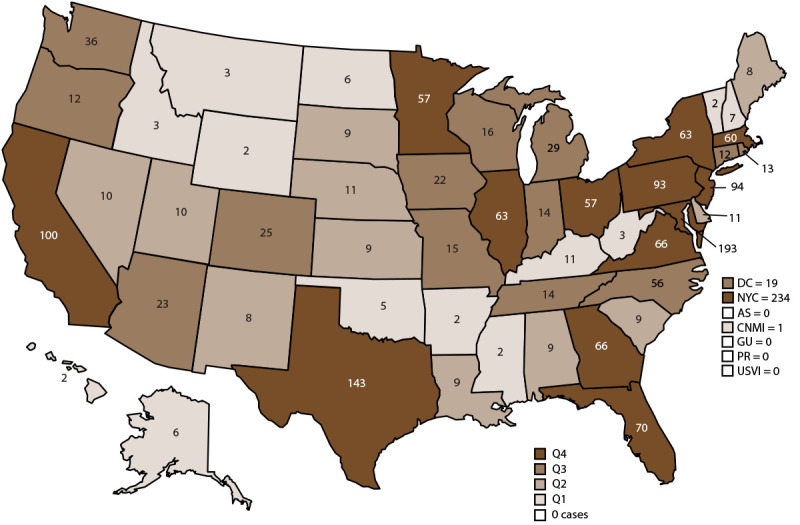
Number of malaria cases,* by state and quartile^†^ — United States, 2018 **Abbreviations:** AS = American Samoa; CNMI = Commonwealth of the Northern Mariana Islands; DC = Washington, DC; GU = Guam; NYC = New York City; PR = Puerto Rico; Q = quartile; USVI = U.S. Virgin Islands. * N = 1,823. New York state cases do not include those from New York City. ^†^ States reporting one or more cases in 2018 were categorized into quartiles: Q1 = 1–7 cases (13 states); Q2 = 8–11 cases (12 states); Q3 = 12–56 cases (14 states); and Q4 = 57 or more cases (14 states).

Confirmed malaria cases were classified according to location of diagnosis or residence of the infected person. All U.S. states and reporting jurisdictions (e.g., New York City and Washington, DC) reported at least two malaria cases in 2018; in addition, the Commonwealth of the Northern Mariana Islands reported one malaria case. States and territories reporting one or more cases were categorized into quartiles (Figure 3). The 14 states in the upper quartile account for 74.5% of cases (1,359 cases) and include New York City (234 cases), Maryland (193 cases), Texas (143 cases), California (100 cases), New Jersey (94 cases), Pennsylvania (93 cases), Florida (70 cases), Virginia (66 cases), Georgia (66 cases), New York State (63 cases [not including New York City]), Illinois (63 cases), Massachusetts (60 cases), Ohio (57 cases), and Minnesota (57 cases). Thirteen of 14 states in the upper quartile were the same in 2017 and 2018; in 2018, Illinois gained 10 cases compared with 2017 (53 cases) and was included in the upper quartile for this year.

### Chemoprophylaxis Use Among U.S. Residents

Among 1,788 imported malaria cases in 2018, a total of 1,102 were U.S residents (civilian and military personnel), and information about chemoprophylaxis use was reported for 974 (88.4%). Approximately 735 (75%) of these patients reported not taking any malaria chemoprophylaxis medication, a larger proportion than what was observed in 2017 (829 [71.7%] of 1,157 U.S. residents with data on prophylaxis use). Altogether, among the 864 U.S. resident imported cases with complete information on chemoprophylaxis, 43 (5.0%) were adherent to an appropriate regimen, 745 (86.2%) did not take chemoprophylaxis or took a regimen that was not consistent with CDC treatment guidelines, and 76 (8.8%) took an appropriate prevention medication but missed doses. The proportion of U.S. resident cases who were adherent to a correct chemoprophylaxis regimen in 2018 was similar to that observed in 2017 (67 patients [6.7%]).

Although approximately equal numbers of the 239 U.S. resident patients who took any chemoprophylaxis were VFR travelers (120 patients [50.2%]) or persons who traveled for other reasons (119 patients [49.8%]), a lower proportion of VFR travelers (17.7% of 677 patients) reported taking any prophylaxis compared with non-VFR travelers (28.0% of 425 patients). Of the 239 patients who reported taking any chemoprophylaxis for preventing malaria during travel, 146 reports provided the name of the antimalarial taken; 48 (32.9%) took doxycycline, 42 (28.8%) took atovaquone-proguanil, 32 (21.9%) took mefloquine, seven (4.8%) took chloroquine, three (2.1%) took primaquine, and 14 (9.6%) took two or more antimalarials for prophylaxis. Among all patients who reported taking any prophylaxis, 200 (83.7%) provided information on adherence; 133 (66.5%) patients did not take all doses, and of these, 89 (66.9%) provided at least one reason why chemoprophylaxis doses were missed. Multiple reasons for missing chemoprophylaxis medications could be provided for each patient, and these reasons included forgetting to take the medication (30 patients [33.7%]); prematurely stopping prophylaxis after returning home from travel (24 patients [27.0%]); running out of the medication during travel (16 patients [18.0%]); incorrectly taking the medication (nine patients [10.1%]); reporting that the prophylaxis was not needed (seven patients [7.9%]); having lost, stolen or destroyed medication (four patients [4.5%]); being advised by others to stop (two patients [2.2%]); and other reasons (four patients [4.5%]). Chemoprophylaxis regimens differ in the requirement to adhere to them after travel is concluded; doxycycline and mefloquine should be taken for 28 days after return, and atovaquone-proguanil should be taken for 7 days after return (*34*). Of the 24 persons who reported missing doses of chemoprophylaxis because they stopped it prematurely after travel, the name of the chemoprophylaxis medication was reported for 19 (79.2%) persons. Regimens that had longer posttravel requirements were more commonly stopped prematurely; 10 (52.6%) patients took doxycycline alone, four (21.1%) patients took mefloquine alone, two (10.5%) patients took doxycycline or mefloquine along with primaquine, and three (15.8%) patients took atovaquone-proguanil.

Of 146 patients who reported the name of the chemoprophylaxis antimalarial taken to prevent malaria, 144 (98.6%) also had the country of acquisition reported so that the appropriateness of the regimen could be ascertained. Of these, 134 (93.1%) reported taking an antimalarial that was correct for the region of travel and 10 (6.9%) did not take a recommended medication. Of 134 patients who took a correct prophylaxis regimen for the region of travel, adherence information was reported for 119 (88.8%) patients and 76 (63.9%) reported missing doses.

Of the 43 persons who reported taking all doses of an appropriate chemoprophylaxis regimen, 23 (53.5%) had *P. falciparum* infections, 11 (25.6%) *P. vivax*, five (11.6%) *P. ovale*, two (4.7%) *P. malariae*, and two (4.7%) an undetermined species. Primary prophylaxis can prevent acute illness of *P. vivax* and *P. ovale* infections, although patients might experience a relapsing infection unless primaquine was taken to eliminate dormant hypnozoites (radical cure). For the 16 patients who reported adherence to chemoprophylaxis and were infected with *P. vivax* or *P. ovale* parasites, seven patients had traveled in Asia; six in Africa; and one each in Central America and the Caribbean, South America, and Oceania. Of these 16 patients, five took mefloquine for prophylaxis, four took atovaquone-proguanil, two took doxycycline, and five patients took two or more antimalarials for chemoprophylaxis. The time between return to the United States and onset was available for 14 of 16 patients with *P. vivax* or *P. ovale* infections; of these, 11 (78.6%) had onset dates more than 45 days from return, suggesting that the infection could be consistent with a relapsing illness.

Of the 25 patients who reported complete adherence to an appropriate chemoprophylaxis regimen and were infected with *P. falciparum* (n = 23) and *P. malariae* (n = 2), all had traveled to Africa. Twenty-one had information on the interval between the return date and onset of illness; of these, 20 (95.2%) had illness onset within 30 days of return to the United States, nine took atovaquone-proguanil, seven took doxycycline or mefloquine, and two took two or more antimalarials for prevention.

In 2018, specimens were not tested for molecular markers of resistance for any of the patients who reported adherence to chemoprophylaxis. Possible explanations for nonrelapsing *Plasmodium* infections (e.g., *P. falciparum* or *P. malariae*) in patients who adhered to chemoprophylaxis include inadequate dosing or malabsorption of the medication, inaccurate reporting of adherence, emerging parasite resistance, or chemoprophylaxis failure.

#### Uncomplicated Malaria

Among the 1,823 confirmed malaria cases in 2018, a total of 1,572 (86.2%) were uncomplicated, a similar proportion to 2017 (85.6%). Treatment information was reported for 1,296 (82.4%) of cases with uncomplicated malaria, and the most common antimalarial treatment administered was atovaquone-proguanil, given to 789 (60.9%) of 1,296 patients with medications recorded, followed by artemether-lumefantrine for 213 (16.4%) patients. A quinine regimen was recorded for 159 (12.3%) patients, chloroquine for 91 (7.0%) patients, and mefloquine for 40 (3.1%) patients.

Among 1,572 patients with uncomplicated malaria, 988 (62.8%) were hospitalized for their illness. Of the 1,059 patients with uncomplicated *P. falciparum* infections, 700 (66.1%) were hospitalized; of the 165 patients with uncomplicated *P. vivax* infections, 115 (69.7%) were hospitalized; of the 88 patients with uncomplicated *P. ovale* infections, 61 (69.3%) were hospitalized; of the 45 patients with uncomplicated *P. malariae* infections, 23 (51.1%) were hospitalized; of the 15 patients with uncomplicated mixed species infections, five (33.3%) were hospitalized; and of the 199 patients with unknown species of malaria, 84 (42.2%) were hospitalized. One patient with an uncomplicated *P. knowlesi* infection was not hospitalized.

The CDC Guidelines for Treatment of Malaria in the United States, herein referred to as the CDC guidelines, provide guidance for the treatment of malaria according to species, disease severity, pregnancy status, and region of acquisition (*32*). Of the 1,572 patients with uncomplicated malaria, 1,282 (81.6%) patients had records containing treatment and travel information; appropriate treatment was given to 1,176 (91.7%) patients. Of 1,032 patients with uncomplicated *P. falciparum* illness, information was available to determine appropriateness of treatment for 911 (88.3%), and treatment was given according to guidelines for 861 (94.5%) patients. In 2018, there were 45 patients with uncomplicated *P. malariae* and one with *P. knowlesi* infections; among these 46 patients, sufficient information was recorded for 37 (80.4%), and appropriate treatment was given to 35 (94.6%).

Among 281 patients with confirmed *P. vivax* or *P. ovale* infections (including 13 mixed infections with *P. vivax* or *P. ovale*), 265 (94.3%) of these were uncomplicated; one patient with a *P. vivax* infection was pregnant. In addition to an antimalarial for the acute infection, an 8-aminoquinoline (in 2018 primaquine was the only option) is recommended to prevent *P. vivax* or *P. ovale* relapse among persons who are not pregnant and who are glucose-6-phosphate dehydrogenase (G6PD) competent. Of the 265 patients with uncomplicated *P. vivax* or *P. ovale* species infections, treatment and travel data were available for 225 (84.9%). Appropriate treatment for the acute illness was given to 194 (86.2%) patients with uncomplicated infections with *P. vivax* or *P. ovale*. Primaquine to prevent relapsing illness because of dormant hypnozoites was reported as treatment for 102 (52.6%) of these patients. A total of 137 patients with uncomplicated malaria were given primaquine; 18 persons with *P. falciparum* or *P. malariae* infections had documented primaquine treatment, which is not recommended. Seventeen patients with unknown species determination were given primaquine.

Of 1,572 patients with uncomplicated malaria, 223 (14.2%) reported a history of malaria in the previous 12 months, a similar proportion to what was observed in 2017 (280 [15.1%] of 1,849 patients with uncomplicated malaria). The species for the current, acute illness was reported for 206 (92.4%) of these patients, and 117 (56.8%) were *P. falciparum*, 59 (28.6%) were *P. vivax*, 16 (7.8%) were *P. ovale*, and seven (3.4%) each were *P. malariae* and mixed species infections.

#### Severe Malaria

In 2018, there were 251 (13.8%) patients with severe malaria; this is similar to 2017, when 312 (14.4%) patients had severe malaria. Seven patients with severe illness died from malaria (2.8%) and all were infected with *P. falciparum*. The patient with an induced malaria infection (acquired from a bone marrow transplant) and the patient with a cryptic infection (where the origin was not determined) had severe malaria. A total of 177 (70.5%) patients with severe malaria were adults aged 18–64 years; 48 (19.1%) were infants, children, and adolescents aged <18 years, and 26 (10.4%) were adults aged ≥65 years. Of the 48 pediatric patients with severe malaria, 12 (25.0%) were aged <5 years. Of all 1,397 adults aged 18–64 years with malaria, 12.7% had severe malaria; this is a lower proportion than the 132 adults aged ≥65 years, of which 19.7% had severe malaria. Of the 294 infants, children, and adolescents aged <18 years with malaria, 16.3% had severe malaria; of the 66 infants and children aged <5 years with malaria, 18.2% had severe malaria. These proportions were similar to adults aged 18–64 years. Four patients with severe malaria were pregnant.

Among the 251 patients with severe malaria, the species was determined for 234 (93.2%). A total of 214 persons (91.5%) had *P. falciparum* infections, eight (3.4%) had *P. vivax*, seven (3.0%) had *P. ovale*, and approximately 1% had *P. malariae* (3 [1.3%]) or mixed species (2 [0.9%]) infections. The residence status was recorded for 218 (86.9%) of 251 severe cases, and 174 (79.8%) patients were civilian U.S. residents, four (1.8%) were U.S. military patients, and 40 (18.3%) were non-U.S. residents. Among the 1,066 U.S. civilians with malaria, a higher proportion had severe cases (174 [16.3%]) compared with non-U.S. residents (40 of 375 [10.7%]). The residence status was recorded for six of the seven patients with fatal cases, and all were among U.S. civilians.

Of the 176 U.S. residents with imported severe malaria (civilian and military), chemoprophylaxis use was reported for 165 (93.8%), and 138 (83.6%) reported taking no chemoprophylaxis medication for malaria prevention before, during, or after travel. Of the 27 who reported taking any chemoprophylaxis, 10 did not provide information on the regimen or adherence, 14 took a regimen that was not consistent with CDC guidelines or missed doses of a correct regimen, and three reported taking all doses of a correct regimen. Of the three patients with severe malaria who adhered to a recommended chemoprophylaxis regimen, two took atovaquone-proguanil, and one took doxycycline. Potential reasons for chemoprophylaxis failure in these patients with severe malaria include inadequate dosing or malabsorption of the chemoprophylaxis medication, inaccurate reporting of adherence, or emerging parasite resistance.

Patients with severe malaria can have multiple clinical complications. Among patients with severe malaria in 2018, acute kidney injury was the most common complication and was experienced by 38 (15.1%) patients, cerebral malaria by 34 (13.5%) patients, acute respiratory distress syndrome by 22 (8.8%) patients, and severe anemia by 22 (8.8%) patients. Parasitemia was reported for 196 (78.1%) of 251 patients with severe malaria, and 134 (68.4%) of these had ≥5.0% parasitized red blood cells. CDC guidelines in 2018 stated that patients with severe malaria should be treated aggressively in an inpatient setting with IV quinidine gluconate or artesunate. Hospitalization status was reported for 250 (99.6%) severe cases, and 235 (94.0%) of these patients were hospitalized. Of the 251 patients with severe malaria, treatment information was available for 242 (96.4%), and parenteral treatment was administered to 151 (62.4%). Of the 151 patients who received parenteral treatment, 118 (78.1%) received quinidine gluconate, 21 patients received artesunate (13.9%), and 12 patients received both quinidine gluconate and artesunate (7.9%). Sufficient treatment and travel information was available for 238 (94.8%) of 251 patients with severe malaria to assess appropriateness of treatment, and 124 (52.1%) of patients with severe malaria did not receive treatment according to CDC guidelines; this is similar to what was observed in 2017 (45.2% of patients with severe malaria received inappropriate treatment). Patients with severe malaria were more likely to have inappropriate treatment (124 of 238 [52.1%]) compared with those with uncomplicated malaria (106 [8.3%] of 1,277).

Of 251 patients with severe malaria, 21 (8.4%) reported a history of malaria during the previous 12 months. This is a similar proportion to what was observed in 2017 (35 [11.2%] of 312 patients with severe malaria had a history of malaria). The species for the current, acute illness was reported for 19 of 21 patients with a history of malaria, and 11 of these were *P. falciparum* (57.9%), four (21.1%) were *P. ovale*, two (10.5%) were *P. vivax* and one each (5.3%) were *P. malariae* and mixed species infection. Among the 234 patients with severe malaria and the current infecting species determined, a higher proportion of patients with non-*falciparum* infections (eight of 20 [40.0%]) had recent illness compared with 11 (5.1%) of 214 patients with a current *P. falciparum* infection.

#### Special Populations

**Military personnel**. Among the 38 cases of malaria confirmed in U.S. military personnel, 25 cases were acquired from Asia (19 cases from Afghanistan and six cases from South Korea), 12 were from Africa (three cases from Africa, unspecified; five from West Africa; three from Eastern Africa; and one from Central Africa); and the country of acquisition was not reported for one case. Four military patients acquired malaria after traveling to Africa to visit friends and relatives, and travel was not deployment related. Twenty-five (65.8%) U.S. military personnel reported taking any prophylaxis; of these, seven (28.0%) took all doses of a correct regimen. Eleven *P. falciparum* and one *P. ovale* infections were acquired by U.S. military members who had traveled to Africa. Of the 25 military patients who had traveled to Asia, 24 infections were confirmed as *P. vivax*, and one patient did not have the species confirmed. The one military patient for whom the country of origin was unknown was infected with *P. falciparum*. Of the 25 military personnel who acquired malaria in Asia, complete information on the date of return to the United States after travel and the onset date was available for 22, and the mean time from the travel return date to symptom onset was 186.6 days. Four U.S. military patients had severe illness, two with *P. falciparum* acquired in Africa or an unknown country, and two with *P. vivax* acquired in Afghanistan. All four military patients with severe illness were treated with IV medication for severe malaria (three with quinidine and one with artesunate). All U.S. military patients recovered from their illness.

**Pregnant women.** Of 683 women, 19 (2.8%) were pregnant, 15 had uncomplicated illness, and four pregnant women had severe malaria. All pregnant women acquired malaria in Africa; 15 were *P. falciparum*, one was *P. vivax*, and three had infections where the species was not determined. The residence status for all pregnant women was reported, and 11 were U.S. residents (nonmilitary). Of the 11 pregnant women who were U.S. residents, nine traveled to visit friends and relatives, one for tourism, and one for an unknown reason. Of the eight non-U.S. residents who traveled to the United States, four were refugees or immigrants, two visited friends or family, and one each traveled to the United States for a medical reason or for an unknown reason. Five pregnant women reported having a history of malaria in the 12 months before the current illness, and four of them were non-U.S. residents.

Antimalarial drug choices to prevent or treat malaria during pregnancy are limited because of the safety profile of medications approved for pregnancy and drug resistance patterns. In most areas where malaria is endemic, only mefloquine is approved for chemoprophylaxis; until 2018, only mefloquine or quinine with clindamycin was recommended as treatment for uncomplicated malaria during pregnancy (*32*). Chloroquine for chemoprophylaxis or treatment of uncomplicated malaria is recommended only for pregnant women to use when their travel is within limited geographical areas where chloroquine-sensitive malaria is transmitted. Primaquine can cause hemolytic anemia among persons with G6PD deficiency and should not be administered during pregnancy. Of 11 pregnant women who were U.S. residents, 10 provided information on chemoprophylaxis use, nine did not take chemoprophylaxis to prevent malaria during travel, and one reported adhering to an unnamed medication for the prevention of malaria.

Of the 15 pregnant women with uncomplicated malaria, two were missing treatment information. Among the 13 pregnant women with uncomplicated malaria and sufficient information to assess the appropriateness of treatment, six were treated according to CDC guidelines (four with quinine and clindamycin and two with mefloquine). In April 2018, the treatment guidelines changed to recommend artemether-lumefantrine for pregnant women in the second or third trimester or in the first trimester if other options are not available and the benefits outweigh the risks (*54*). Five pregnant women with uncomplicated malaria were treated with artemether-lumefantrine in 2018; three had onset dates after the guidelines changed, and two were treated with artemether-lumefantrine before April 2018. The gestational age of the pregnancy was not documented on the malaria case report for any of the women receiving artemether-lumefantrine, making it difficult to assess the appropriateness of treatment for those treated after the update in recommendations. Two women with *P. falciparum* infections were administered inappropriate treatment; one was treated with atovaquone-proguanil and one with chloroquine and clindamycin. None of the pregnant women with uncomplicated malaria were administered primaquine.

Severe malaria in pregnant women should be treated aggressively with IV regimens; in the United States during 2018, the treatment options for severe malaria were quinidine-gluconate or artesunate. Three of the four patients with severe malaria were not treated according to guidelines. Two pregnant women with severe malaria were treated with oral medications only, and two women were treated with a parenteral antimalarial (quinidine-gluconate). Although pregnancy outcomes are not known, all pregnant women with severe malaria recovered, and no case of congenital malaria was reported in 2018.

### Drug Resistance Markers

CDC received 154 (8.4%) whole blood specimens for molecular surveillance from patients who received a malaria diagnosis in 2018. A total of 43 of these specimens were PCR confirmed as *P. vivax* (n = 16), *P. ovale* (n = 17), *P. malariae* (n = 7), or as *Plasmodium* genus (n = 3); no molecular resistance testing was performed. For one specimen that was PCR confirmed as a mixed infection with *P. falciparum* and *P. ovale*, no amplification of resistance markers was achieved because of a low level of parasitemia. A total of 110 specimens, including 106 *P. falciparum* and four mixed species infections (three *P. falciparum* and *P. malariae* and one *P. falciparum* and *P. ovale*) underwent amplification and analysis of resistance mutations for at least one antimalarial drug (Table 8). The region of acquisition was known for 108 (98.2%) of 110 specimens tested for antimalarial resistance; of these, 107 (99.1%) were acquired from Africa and one (<1%) from Asia.

**TABLE 8:** Antimalarial drug resistance marker results among *Plasmodium falciparum* specimens, by drug and region of malaria acquisition — United States, 2018

Resistance markers	Region			
	Africa No. (%)	Asia No. (%)	Unknown* No. (%)	Total No. (%)
**Pyrimethamine**	**98 (97.0)**	**1 (1.0)**	**2 (2.0)**	**101 (100)**
No resistance markers	2 (2.0)	0 (0)	0 (0)	**2 (2.0)**
1 resistance marker	0 (0)	0 (0)	0 (0)	**0 (0)**
2 resistance markers	5 (5.0)	1 (1.0)	0 (0)	**6 (5.9)**
3 or more resistance markers	91 (90.1)	0 (0)	2 (2.0)	**93 (92.1)**
**Sulfadoxine**	**100 (97.1)**	**1 (1.0)**	**2 (1.9)**	**103 (100)**
No resistance markers	52 (50.5)	1 (1.0)	1 (1.0)	**54 (52.4)**
1 resistance marker	26 (25.2)	0 (0)	1 (1.0)	**27 (26.2)**
2 resistance markers	15 (14.6)	0 (0)	0 (0)	**15 (14.6)**
3 or more resistance markers	7 (6.8)	0 (0)	0 (0)	**7 (6.8)**
**Chloroquine**	**107 (97.3)**	**1 (0.9)**	**2 (1.8)**	**110 (100)**
No resistance markers	58 (52.7)	0 (0)	2 (1.8)	**60 (54.5)**
1 resistance marker	0 (0)	0 (0)	0 (0)	**0 (0)**
2 resistance markers	0 (0)	1 (0.9)	0 (0)	**1 (0.9)**
3 resistance markers	49 (44.6)	0 (0)	0 (0)	**49 (44.5)**
**Mefloquine**	**97 (98.0)**	**1 (1.0)**	**1 (1.0)**	**99 (100)**
No resistance markers	95 (96.0)	1 (1.0)	1 (1.0)	**97 (98.0)**
1 resistance marker	2 (2.0)	0 (0)	0 (0)	**2 (2.0)**
**Atovaquone**	**94 (97.9)**	**1 (1.0)**	**1 (1.0)**	**96 (100)**
No resistance markers	94 (97.9)	1 (1.0)	1 (1.0)	**96 (100)**
**Artemisinin**	**107 (97.3)**	**1 (0.9)**	**2 (1.8)**	**110 (100)**
No resistance markers	107 (97.3)	1 (0.9)	2 (1.8)	**110 (100)**

* There were no specimens for molecular resistance marker analysis acquired from South America, Central America and the Caribbean, or Oceania.

Of the 101 (98.2%) specimens that amplified for five pyrimethamine loci, two specimens (acquired from Africa) had no resistance markers, six (5.9%) had two resistance markers, and 93 (92.1%) had three or more resistance markers. A total of 103 specimens (96.6%) had five sulfadoxine resistance loci analyzed; 54 (52.4%) of these had no markers of resistance, 27 (26.2%) had one resistance marker, 15 (14.6%) had two resistance markers, and seven (6.8%) had three or more resistance marker mutations. All 110 specimens were examined for chloroquine resistance at five loci. No chloroquine resistant markers were identified for 60 (54.5%) specimens, one (<1%) specimen (from Asia) had two resistant markers, and 49 (44.5%) had three chloroquine resistance markers. Copy number variation of the mefloquine locus (*pfmdr1*) is associated with mefloquine resistance, and 99 (90.0%) specimens were examined; two (2.0%) specimens from Africa had more than one copy of *pfmdr1.* The genes associated with atovaquone and artemisinin (*pfk13*) resistance were evaluated in 96 (87.3%) and 110 (100%) specimens, respectively, and no markers of resistance were identified for either antimalarial.

Because of widespread resistance to pyrimethamine and sulfadoxine, CDC does not recommend using drugs containing these components to treat patients with malaria in the United States (*32*). All patients with chloroquine resistance markers had exposure to malaria in Africa or Asia, which are considered regions with chloroquine resistance, so this antimalarial is not recommended as treatment for these patients. The two patients with multiple copies of the mefloquine (*pfmdr1*) gene had traveled to Ivory Coast and Liberia to visit friends and relatives, both had uncomplicated malaria, and both were treated appropriately with atovaquone-proguanil and recovered; no prophylaxis use was reported for either patient.

### Selected Malaria Case Reports

#### Cryptic Case

**Patient.** In early October, an otherwise healthy man aged 43 years developed onset of fever, weakness, arthralgias, and dark urine. Four days later, he was admitted to the hospital and received a diagnosis of severe *P. falciparum* malaria with 10.7% parasitemia and acute kidney injury. Quinidine was not available at the hospital or in the surrounding area, and he was started on an oral antimalarial regimen while artesunate was requested from CDC. The morning of his second day of admission, artesunate treatment was initiated and followed by oral atovaquone-proguanil, and the patient recovered.

**Investigation.** Patient blood specimens were sent to CDC, and PCR confirmed *P. falciparum* infection. The patient was interviewed twice, once by the health care provider and a second time by the state health department. The man was born in Ghana but had not lived there since 1995; he moved to the United States from Germany in 2008. His most recent international travel to a country where malaria is endemic was approximately 10 years earlier, and he denied having a recent history of malaria. The patient did not agree to a passport review, and no official travel documents could be verified. The patient did not report exposure to blood products, IV drug use, or another unusual exposure. No family or friends had been ill with a febrile illness, and the patient did not have an immunosuppressive condition. To rule out the possibility of local transmission, the state health department identified two additional patients who had received diagnoses of *P. falciparum* malaria with geographical and temporal proximity to the patient with cryptic malaria. One patient who lived approximately 20 miles away was diagnosed with *P. falciparum* that was acquired from travel to Togo 7 weeks before the patient with cryptic malaria had symptom onset. The other patient with *P. falciparum* had symptom onset approximately 4 weeks before the symptom onset of the patient with cryptic malaria and lived 6 miles from the patient with the cryptic case; this infection was acquired in Tanzania. Specimens from all three cases were submitted to CDC for PCR species confirmation and genetic characterization to determine parasite relatedness; drug resistance markers were evaluated, and neutral microsatellite analysis was conducted. Mutations observed in the *pfcrt*, *pfdhps*, *pfdhfr,* and *pfmdr1* genes from all specimens were indicative of parasites acquired in Africa. Seven neutral microsatellites were examined, and the three specimens had different microsatellite profiles indicating that the parasites were unrelated. County mosquito surveillance data were reviewed to assess the possibility of local mosquitoborne transmission, but there were no *Anopheles* mosquitos trapped during the 2018 summer season in the county where the patient resides. It is unlikely that this case-patient with cryptic malaria acquired *P. falciparum* infection through local mosquitoborne transmission. Possible explanations for this cryptic case include nondisclosed travel or, less likely after 10 years, recurrence of malaria from late emergence of asymptomatic parasitemia.

#### Induced Case

**Recipient.** A woman aged 24 years with sickle cell disease received a familial bone marrow transplant (BMT). Fifteen days after the transplant, she experienced fever, and the following day *Plasmodium* parasites with 10% parasitemia were identified on a routine blood smear. The patient had no clinical complications documented, was treated with parenteral quinidine-gluconate, doxycycline, and atovaquone-proguanil, and recovered. CDC confirmed *P. falciparum* infection by microscopy and PCR.

**Investigation.** The recipient had never traveled to an area where malaria is endemic nor had she experienced a previous malaria illness. The BMT donor had traveled to Ghana 1.5 years before donation. Upon returning from travel, the donor reported malaria-like symptoms that eventually resolved. Blood smears at the time were negative, and the donor was not treated for malaria.

Donor specimens collected after the BMT were tested by CDC. No *Plasmodium* parasites were detected in the donor blood smear. A donor whole blood specimen was positive for *P. falciparum* by real-time PET-PCR with a cycle threshold value of 38, which is close to the threshold for positivity (40), indicating a low level of parasitemia. The donor was also positive for *Plasmodium* genus and *P. falciparum* antibodies, but negative for parasite antigens. Microsatellite genotype testing was performed on both recipient and donor specimens; however, this was successful only for the recipient specimen so the genetic signatures could not be compared. The investigation concluded that the recipient acquired a *P. falciparum* infection after a BMT from a donor with asymptomatic parasitemia.

#### Seven Fatal Cases

Six men and one woman died from severe malaria in the United States in 2018. Their mean age was 59.1 years; the youngest was aged 41 years and the oldest was aged 72 years. Three were Black or African American, three were White (one of whom was of Hispanic or Latino), and one had an unreported race. The residence status was reported for six of seven fatal cases, and all were among U.S. residents. All had traveled to countries in Africa and were infected with *P. falciparum*. The reason for travel was reported for six patients; three had traveled for business, one for tourism, one for education purposes, and one to visit friends or relatives. All patients sought care for their symptoms (range: within 2–14 days after symptoms onset), and six were hospitalized; the mean time from symptom onset to hospital admission was 7.2 days (range: 2–14 days). Two patients sought care 2–3 days after symptom onset, but received diagnoses of influenza, were discharged, and did not receive a prompt workup for malaria. One of these patients was found deceased at home 6 days after the first medical evaluation and received a diagnosis of malaria upon autopsy. Parasitemia was reported for five of seven patients, with a mean of 17.5% (range: 4%–30%). Clinical complications were reported for six cases and included renal failure (five patients), cerebral malaria (four patients), and acute respiratory distress syndrome (one patient). Antimalarial medication was administered to six patients: one was treated with oral medication alone, two were treated with quinidine-gluconate, one was treated with artesunate, and two were treated with both quinidine-gluconate and artesunate. Each fatal case had delayed diagnosis, delayed treatment, or inappropriate treatment for severe malaria. Four patients received appropriate parenteral treatment for severe malaria, but initial care seeking was delayed by 5–14 days. One patient sought care within 2 days of symptom onset, but the diagnosis of malaria was missed, and the patient died without further care. One patient sought care from a primary care provider within 3 days of symptom onset, but the diagnosis of malaria was missed, and the patient did not receive a diagnosis until 3 days later when brought to the emergency department in poor condition 6 days after symptom onset; appropriate treatment was given, but the patient died. One patient sought care within 2 days of symptom onset and received inappropriate (oral antimalarial) treatment for severe malaria.

## Discussion

Since the mid-1970s, an increasing number of U.S. civilians received diagnoses of malaria in the United States, while the number of cases among U.S. military personnel and non-U.S. residents has not increased (Table 2 and Figure 1). The increase in malaria cases coincides with the increasing trend in the annual number of international airline flights taken by U.S. citizens (*55*). In contrast to non-U.S. residents who might live in endemic areas, malaria among U.S. residents is preventable with compliance to chemoprophylaxis. For most military deployments to areas where malaria is endemic, except for the Republic of South Korea (*8*,*56*), there are mandatory chemoprophylaxis measures that promote adherence, and the low number of malaria cases in recent years among this group demonstrates the success of these measures to prevent malaria, even in areas of high malaria transmission (*57*,*58*). In addition to taking certain mosquito avoidance measures (*23*,*59*), adhering to an antimalarial chemoprophylaxis regimen is the best way for travelers to prevent this potentially life-threatening illness. Chemoprophylaxis should be appropriate for the traveler’s age, pregnancy status, destination country, preferences (e.g., daily or weekly drug regimen), and potential medication side effects.

In 2018, as in previous years, approximately 60% of all cases imported into the United States were attributable to persons who had traveled in West Africa, and two thirds of VFR travelers went to West Africa. This large contribution of imported malaria among VFR travelers from West Africa has been observed in other settings (*60*–*63*). Changes in travel because of disease outbreaks, like 2015–2017 during and after the Ebola virus disease outbreak in West Africa where travel restrictions were implemented (*64*), can result in fewer U.S. numbers of malaria imported cases during the time of travel restriction and an increase in cases subsequent to the disease outbreak when persons might travel at similar times after the interruption ceases (*51*,*55*,*65*). Likewise, the hypothesis is that the COVID-19 pandemic might result in an initial decrease in cases while recommendations to limit travel are in place and perhaps an increase in cases compared with previous years after travel limitations are removed.

Among U.S. civilians with malaria, the most common reason for travel is VFR (*51*,*55*,*65*–*67*). However, among those with malaria, VFR travelers were less likely to take chemoprophylaxis to prevent malaria (17.7% among VFR travelers versus 28.0% among non-VFR travelers). Focus group studies and antimalarial pricing studies identified complex barriers that might be encountered by VFR travelers to obtain and adhere to chemoprophylaxis (*68*–*72*). Although one of the identified barriers is an incorrect perception of being low risk for acquiring malaria or having severe illness, the other barriers identified deal with care seeking and access to pretravel care. In the United States, obtaining malaria prophylaxis involves a consultation and prescription from a health care provider, which requires knowledge about the need for pretravel care, and having the time and financial resources to pay for the visit and medications, all potential challenges for the VFR traveler. Additionally, the cost of antimalarials varies widely, and even if the patient has insurance, the insurance might not cover any or all of the medications because of dispensing limitations (*68*,*72*,*73*); VFR travelers often visit an endemic area for a long duration and might require more than a 1-month supply. Also, during travel they might face pressure not to inconvenience their hosts with prevention measures, possibly resulting in lower adherence (*69*–*71*).

Among those with malaria and information on chemoprophylaxis adherence, stopping a recommended chemoprophylaxis regimen prematurely upon completion of travel was given as a reason for nonadherence. Chemoprophylaxis regimens that need to be taken for a shorter duration after travel might improve adherence (*74*). In 2018, FDA approved tafenoquine (Arakoda) for malaria chemoprophylaxis in nonpregnant women and adults aged ≥18 years who are not G6PD deficient as confirmed by a quantitative G6PD test result (*75*). Arakoda chemoprophylaxis offers protection from acute *Plasmodium* infections, in addition to protection from relapsing infections. Although Arakoda chemoprophylaxis offers a potentially convenient and abbreviated dosage schedule (including a single dose during the week after departing the area where malaria is endemic) and protection from relapsing malaria infections, use of Arakoda requires enough time to acquire pretravel care and G6PD testing results (*74*).

Malaria is a medical emergency. Prompt diagnosis and treatment of malaria is necessary to prevent severe disease or death. All species of *Plasmodium* can cause severe illness, and any delay in the diagnosis and treatment of malaria can result in complications, regardless of the effectiveness of the treatment regimen. In 2018, all seven patients who died experienced a delay in the diagnosis of or proper treatment for malaria; four patients did not promptly seek care for their illness and died despite receiving appropriate treatment for severe malaria, two patients were not diagnosed with malaria during the initial provider consultation, and one patient with a prompt diagnosis of malaria was given oral antimalarials (an inappropriate treatment) for severe illness. Malaria is the most common single etiology of fever in the returned traveler (*76*,*77*). With international travel increasing over the past decades, returning travelers with fevers will increasingly be seen by front-line health workers who should be prepared to diagnose or rule out malaria rapidly.

Travelers should be educated about the importance of seeking care as soon as possible if fever develops after travel in a country where malaria is endemic and told to notify care providers of their travel history. Although malaria symptoms typically occur within 3 months of travel, *P. vivax* and *P. ovale* cases can have a delayed initial onset of 6 months or more after travel; this delay in symptom onset is likely because of relapsing infections commonly associated with these species. In this report, a delay in symptom onset was particularly relevant for military personnel who were deployed to Afghanistan and South Korea, and due to the drug effect on the parasites, chemoprophylaxis use might contribute to an even later onset of symptoms. To facilitate a prompt diagnosis, providers should ask all febrile patients for a travel history and include malaria in the differential diagnosis of fever in a person who has traveled to a country with malaria transmission or for those who are originally from an area where malaria is endemic. Signs and symptoms of uncomplicated malaria are often nonspecific but typically include fever. Other symptoms include headache, chills, increased sweating, back pain, myalgia, diarrhea, nausea, vomiting, and cough.

Blood smears done and read immediately are the primary test for malaria diagnosis. Thick blood smears are more sensitive than thin blood smears for detecting malaria parasites because a greater volume of blood is examined in each microscopic field. Thin blood smears facilitate identification and quantification of the parasite species (*78*). The percentage of parasitized red blood cells should be calculated and provided promptly to the clinical team so that disease severity and appropriate treatment can be initiated. Health care providers should assess the diagnostic and treatment resources present in their facilities, including availability at night and on weekends. The resources required to perform a malaria blood smear examination are similar to those for the commonly performed complete blood count with a manual differential. Having qualified personnel who can perform this task on call 24 hours per day can facilitate timely diagnosis. Sending specimens to offsite laboratories can incur a delay in results. In 2017, results from a follow-up to a 2010 nationwide survey of laboratories conducted in the United States (*79*,*80*) found that although most laboratories offered malaria diagnostic testing services, only 12% were in complete compliance with all the Clinical and Laboratory Standards Institute guidelines for analysis and reporting of results (https://clsi.org/standards/products/microbiology/documents/m15). Limited laboratory experience with malaria specimens can be a barrier to accurate diagnoses, and three fourths of laboratories surveyed reported diagnosing five or fewer cases annually (*80*). Despite some progress in laboratory performance, opportunities are available to further improve adherence to malaria diagnosis recommendations; specifically, 19% of laboratories did not offer malaria testing at all times, 43% did not return results within 4 hours, and 57% did not return percentage parasitemia within 6 hours (*80*). CDC provides telediagnosis assistance to laboratories and care providers for blood smear diagnosis (*81*) in addition to training resources for malaria and other parasitic diseases (*82*). Clinicians evaluating patients with suspected or confirmed malaria should obtain a timely infectious disease consultation and consider additional assistance from the CDC Malaria Hotline (770-488-7788 or toll-free 855-856-4713 during regular business hours, or CDC’s Emergency Operations Center at 770-488-7100 during evenings, weekends, and holidays). Additional information is available online at https://www.cdc.gov/malaria/diagnosis_treatment/index.html.

Laboratories unable to provide immediate blood smear microscopy should maintain a supply of BinaxNow malaria rapid diagnostic tests (RDTs) to assist with the immediate diagnosis of malaria; however, blood smears should be prepared in parallel, with results available as soon as possible to confirm the RDT results and to provide the percentage of red blood cells infected to aid in determining uncomplicated versus severe disease (*43*). The BinaxNow malaria RDT detects an antigen found in all *Plasmodium* species in addition to the histidine-rich protein-2 (HRP2) antigen found in many *P. falciparum* infections. False negative results are more likely with non-*falciparum* infections (*42*,*43*) and with emerging *P. falciparum* strains that harbor *hrp2* gene deletions; users should be aware of these limitations when administering malaria RDTs (*83*). Empiric treatment for malaria is not recommended because if a patient has malaria, then an assessment of disease severity, which includes the percent parasitemia, is not possible, which could result in improper treatment. If the patient does not have malaria, then failing to diagnose and treat the real reason for illness can lead to poor outcomes because of progression of the underlying illness.

PCR testing should not be relied on for the initial diagnosis of malaria because it does not provide timely results and diagnostic PCRs do not provide information on the level of parasitemia. However, PCR is the most definitive test to determine the infecting species and should be used to confirm the results of blood smear microscopy and evaluate for mixed infections. In 2014, the Council of State and Territorial Epidemiologists released a revised malaria case definition highlighting the importance of determining the species and the percent parasitemia at the time of diagnosis and encouraging PCR testing for each case (*27*). If PCR testing is not available locally to confirm the *Plasmodium* species, then whole blood and blood smears can be sent to state public health laboratories or to CDC for diagnostic confirmation and drug resistance marker analysis (*81*). Increasing the proportion of cases with species confirmation and drug resistance marker analysis will improve the epidemiologic understanding of malaria diagnosed in the United States. Specifically, drug resistance marker surveillance of imported cases might be able to detect changes in the prevalence of drug resistance markers in the countries of malaria acquisition, in which case the U.S. treatment guidelines could be revised. In addition, understanding the regional molecular profiles of parasites acquired worldwide could provide additional evidence that would help explain the malaria cases investigated in the United States, especially cryptic cases of malaria. Public health laboratories should consider developing standardized procedures for sending blood specimens from persons with malaria to CDC for molecular surveillance monitoring.

The choice of an antimalarial treatment regimen should be based on several factors, including the probability of drug resistance based on geographic origin of the parasite, the *Plasmodium* species, the percent parasitemia, and the patient’s pregnancy and clinical status (*32*,*36*). Oral regimens are less effective than parenteral regimens for treatment of severe malaria and are not the standard of care. Severely ill patients should be treated aggressively with parenteral antimalarial therapy to ensure rapid adequate drug levels, with the goal to decrease parasitemia to <1% as soon as possible to minimize the likelihood of complications or death (*32*). Quinidine gluconate was the only FDA-approved medication for parenteral malaria therapy in 2018; however, as of April 2019, quinidine gluconate is no longer available in the United States (*84*). Beginning in April 2019, CDC made IV artesunate, the WHO-recommended first-line drug for severe malaria, available through an investigational new drug (IND) protocol for every patient in the United States with severe malaria (*85*). In 2020, FDA approved an IV artesunate product (*86*,*87*), and the commercial product has been available from major drug distributors for hospitals to procure since March 2021. With the IV artesunate supply and distribution adequate, CDC will discontinue distribution of investigational artesunate under the IND protocol on September 30, 2022 (*88*). An urgent infectious disease consultation can facilitate timely management of malaria, and CDC has resources on malaria prevention and treatment (Table 1). In 2018, as in most previous years, malaria cases were detected in all U.S. states. To give patients with malaria the best chance of a complete recovery and no sequelae, hospitals can develop a plan for rapid malaria diagnosis via blood smear microscopy and malaria RDTs, stock antimalarials like artemether-lumefantrine (Coartem) and IV artesunate for treatment and, if stocking IV artesunate is not possible, there should be a plan for emergency procurement of IV artesunate (*87*).

The findings in this report indicate a need for better knowledge among health care providers on diagnosis and treatment of severe malaria, relapsing malarias, and malaria in pregnancy. In 2018, fewer than one half of patients with severe malaria were treated according to CDC guidelines, whereas 91.7% of patients with uncomplicated malaria were treated appropriately. Less than one half of patients with relapsing species of malaria received antirelapse treatment. Approximately 30% of pregnant women with information available were administered treatment inconsistent with CDC guidelines. CDC malaria treatment guidelines are updated regularly to reflect the most current evidence for efficacy and safety. Clinicians can consult the CDC website (*32*) for the most current treatment guidelines.

Imported malaria accounted for more than 99% of the classified cases of malaria in 2018; one nonimported case was acquired from a familial BMT, and one was investigated as cryptic, with the origin remaining uncertain. During and after international outbreaks of infectious diseases such as Ebola virus disease, Zika virus, and COVID-19, CDC and public health officials investigated potential cryptic malaria cases among patients who did not disclose a complete or accurate travel history. For most of the potential cryptic cases, nondisclosed travel was discovered (CDC, unpublished data, 2022); when possible, a passport review can be helpful to identify nondisclosed travel.

Few cases of malaria in the United States are induced from a blood transfusion or transplant (solid organ, bone marrow, or stem cells). Three cases of malaria from a blood transfusion were reported in 2016 and 2017, the first cases since 2011 (*51*,*65*,*89*). Because of blood supply shortages, in April 2020, FDA updated the malaria blood donor recommendations to reduce deferrals (*90*). Residents of countries where malaria is not endemic will be deferred for 3 months after travel to a country where malaria is endemic as long as they have no signs or symptoms of malaria at the time of deferral. The recommendation that persons who recently resided in countries where malaria is endemic should be deferred for 3 years has not changed. Former residents of countries with malaria transmission who have lived in countries where malaria is not endemic for 3 or more years can be deferred for 3 months after short-term travel if they are free of malaria during this time (*90*).

The Health Resources and Services Administration reported that in 2018, a total of 4,992 unrelated and 4,275 related bone marrow and cord blood transplants were performed in the United States (*91*). In 2018, a BMT recipient became ill with *P. falciparum* parasitemia 15 days after transplant from a familial donor; there were no other recipients affected. Investigation findings confirmed that the BMT donor had an asymptomatic infection with PCR-detectable *P. falciparum* parasites. Although the recipient had severe malaria illness, the patient received prompt and appropriate care and recovered.

Before 2018, the treatment choices for pregnant women with uncomplicated malaria were limited to mefloquine or quinine plus clindamycin. After a systematic review of existing data, in April 2018, CDC recommended artemether-lumefantrine for the treatment of uncomplicated malaria among women in the second and third trimesters of pregnancy (*32*). Artemether-lumefantrine can also be considered during the first trimester when other treatment options are unavailable and when the benefits outweigh the risks (*54*). This recommendation is consistent with WHO treatment guidelines and reflects data on treatment efficacy and birth outcomes (*37*,*92*). In 2018, approximately 30% of pregnant women in the United States received artemether-lumefantrine for treatment. In contrast, a recent evaluation of the evidence for the use of atovaquone-proguanil during pregnancy was inconclusive because of the lack of data on birth outcomes, and therefore remains not recommended for the treatment of malaria in any trimester of pregnancy (*93*,*94*). IV artesunate, the first-line therapy for severe malaria in the United States, is also the recommended treatment for severe malaria in pregnant women regardless of trimester.

Although malaria is not endemic in the United States, malaria causes illness and deaths in this country. Surveillance of malaria, a potentially deadly disease, is a collaboration between health care providers, local and state health departments, CDC, and other U.S. government departments and agencies, and vigilance is important to prevent local cases that could be induced via blood or tissue donation or acquired from local mosquito transmission. Imported cases of malaria can reintroduce *Plasmodium* parasites into receptive areas (*16*–*18*) where the disease is not endemic but potential vectors are present and environmental conditions can support the parasite lifecycle (*15*). Competent *Anopheles* mosquitoes and conditions conducive to malaria transmission continue to exist in the United States (*95*). CDC, state and local health departments, and partners are engaging to improve case surveillance reporting for malaria and other nationally notifiable diseases (*24*,*96*). Regardless of the efficiency of the surveillance system, the quality of malaria surveillance can be limited if the data are incomplete or if definitions are incorrectly used. Incomplete reporting of antimalarial treatments and the infecting species could result in the patient being misclassified for receiving treatment according to CDC guidelines. All sections of the malaria case report form should be completed because they provide information for examining malaria trends used to develop recommendations for malaria treatment and chemoprophylaxis.

The increasing trend of imported malaria parallels an increase in international travel over the decades, making improved use of chemoprophylaxis more critical for the population at highest risk, specifically VFR travelers, and preparedness for rapid diagnosis and treatment of malaria. Efforts to improve chemoprophylaxis use among VFR travelers could include working with VFR traveler communities and their providers to improve their understanding of malaria risk, encourage pretravel care, and other efforts to improve access and adherence to chemoprophylaxis. Improving preparedness to rapidly diagnose and treat malaria could include educating front-line providers on the diagnosis and treatment of malaria and having diagnostic tools and antimalarials, especially artemether-lumefantrine and IV artesunate, in stock at hospitals.

Detailed recommendations for preventing malaria are available online at https://wwwnc.cdc.gov/travel/yellowbook/2020/travel-related-infectious-diseases/malaria. Prevention recommendations tailored for each country are available online at https://www.cdc.gov/malaria/travelers/country_table/a.html. CDC biannually publishes recommendations in *Health Information for International Travel* (commonly referred to as the *Yellow Book*), which is available and updated on the CDC Travelers’ Health website (https://wwwnc.cdc.gov/travel/page/yellowbook-home-2020) (Table 1).

## Footnotes

* The term “United States” includes all U.S. states and territories.

† 45 C.F.R. part 46.102(l)(2), 21 C.F.R. part 56; 42 U.S.C. §241(d); 5 U.S.C. §552a; 44 U.S.C. §3501 et seq.

§ Countries that are considered West Africa include Benin; Burkina Faso; Cape Verde; The Gambia; Ghana; Guinea; Guinea-Bissau; Ivory Coast; Liberia; Mali; Mauritania; Niger; Nigeria; Senegal; Sierra Leone; Togo; and West Africa, unspecified.

## References

[1] World Health Organization. World malaria report 2021. Geneva, Switzerland: World Health Organization; 2021. https://www.who.int/teams/global-malaria-programme/reports/world-malaria-report-2021

[2] World Health Organization. World malaria report 2019. Geneva, Switzerland: World Health Organization; 2019. https://www.who.int/publications/i/item/9789241565721

[3] World Health Organization. WHO recommends groundbreaking malaria vaccine for children at risk. Geneva, Switzerland: World Health Organization; 2021. https://www.who.int/news/item/06-10-2021-who-recommends-groundbreaking-malaria-vaccine-for-children-at-risk

[4] Warrell DA, Gilles HM, eds. Essential malariology. 4th ed. Boca Raton, FL: CRC Press; 2002.

[5] Mahittikorn A, Masangkay FR, Kotepui KU, Milanez GJ, Kotepui M. Comparison of Plasmodium ovale curtisi and Plasmodium ovale wallikeri infections by a meta-analysis approach. Sci Rep 2021;11:6409. 10.1038/s41598-021-85398-w 33742015 PMC7979700

[6] Oh MD, Shin H, Shin D, Clinical features of vivax malaria. Am J Trop Med Hyg 2001;65:143–6. 10.4269/ajtmh.2001.65.143 11508390

[7] Battle KE, Karhunen MS, Bhatt S, Geographical variation in *Plasmodium* vivax relapse. Malar J 2014;13:144. 10.1186/1475-2875-13-144 24731298 PMC4021508

[8] Klein TA, Pacha LA, Lee HC, Plasmodium vivax malaria among US forces Korea in the Republic of Korea, 1993–2007. Mil Med 2009;174:412–8. 10.7205/MILMED-D-01-4608 19485113

[9] Sutherland CJ. Persistent parasitism: the adaptive biology of *malariae* and *ovale* malaria. Trends Parasitol 2016;32:808–19. 10.1016/j.pt.2016.07.001 27480365

[10] Fornace KM, Brock PM, Abidin TR, Environmental risk factors and exposure to the zoonotic malaria parasite Plasmodium knowlesi across northern Sabah, Malaysia: a population–based cross-sectional survey. Lancet Planet Health 2019;3:e179–86. 10.1016/S2542-5196(19)30045-2 31029229 PMC6484808

[11] Davidson G, Chua TH, Cook A, Speldewinde P, Weinstein P. Defining the ecological and evolutionary drivers of Plasmodium knowlesi transmission within a multi-scale framework. Malar J 2019;18:66. 10.1186/s12936-019-2693-2 30849978 PMC6408765

[12] Davidson G, Chua TH, Cook A, Speldewinde P, Weinstein P. The role of ecological linkage mechanisms in Plasmodium knowlesi transmission and spread. EcoHealth 2019;16:594–610. 10.1007/s10393-019-01395-6 30675676

[13] Rajahram GS, Cooper DJ, William T, Grigg MJ, Anstey NM, Barber BE. Deaths from Plasmodium knowlesi malaria: Case series and systematic review. Clin Infect Dis 2019;69:1703–11. 10.1093/cid/ciz011 30624597 PMC6821196

[14] Andrews JM, Quinby GE, Langmuir AD. Malaria eradication in the United States. Am J Public Health Nations Health 1950;40:1405–11. 10.2105/AJPH.40.11.1405 14790040 PMC1528986

[15] Kiszewski A, Mellinger A, Spielman A, Malaney P, Sachs SE, Sachs J. A global index representing the stability of malaria transmission. Am J Trop Med Hyg 2004;70:486–98. 10.4269/ajtmh.2004.70.486 15155980

[16] CDC. Local transmission of Plasmodium vivax malaria—Palm Beach County, Florida, 2003. MMWR Morb Mortal Wkly Rep 2003;52:908–11. 14508439

[17] CDC. Multifocal autochthonous transmission of malaria—Florida, 2003. MMWR Morb Mortal Wkly Rep 2004;53:412–3. 15152184

[18] Filler SJ, MacArthur JR, Parise M, ; CDC. Locally acquired mosquito-transmitted malaria: a guide for investigations in the United States. MMWR Recomm Rep 2006;55(RR-13):1–9. 16960552

[19] Pérez-Mazliah D, Ndungu FM, Aye R, Langhorne J. B-cell memory in malaria: myths and realities. Immunol Rev 2020;293:57–69. 10.1111/imr.12822 31733075 PMC6972598

[20] Rochford R, Kazura J. Introduction: immunity to malaria. Immunol Rev 2020;293:5–7. 10.1111/imr.12831 31863482

[21] CDC. Malaria. Atlanta, GA: US Department of Health and Human Services, CDC; 2020. https://www.cdc.gov/malaria/about/biology/index.html

[22] Leder K, Black J, O’Brien D, Malaria in travelers: a review of the GeoSentinel surveillance network. Clin Infect Dis 2004;39:1104–12. 10.1086/424510 15486832

[23] Brunette GW, Nemhauser JB, eds. Environmental hazards & other noninfectious health risks [Chapter 3]. In: *CDC Yellow Book 2020*: health information for international travel. New York, NY: Oxford University Press; 2017.

[24] CDC. National Notifiable Diseases Surveillance System (NNDSS). Atlanta, GA: US Department of Health and Human Services, CDC; 2022. https://www.cdc.gov/nndss/index.html

[25] CDC. Electronic Laboratory Reporting (ELR). Atlanta, GA: US Department of Health and Human Services, CDC; 2021. https://www.cdc.gov/elr/index.html

[26] CDC. National Notifiable Diseases Surveillance System (NNDSS): how we conduct case surveillance. Atlanta, GA: US Department of Health and Human Services, CDC; 2021. https://www.cdc.gov/nndss/about/conduct.html

[27] Council of State and Territorial Epidemiologists. Public health reporting and national notification for malaria. Atlanta, GA: Council of State and Territorial Epidemiologists; 2014. https://cdn.ymaws.com/www.cste.org/resource/resmgr/PS/13-ID-08.pdf

[28] BinaxNOW MALARIA [package insert]. Abbott Park, IL: Abbott; 2020. https://www.globalpointofcare.abbott/en/product-details/binaxnow-malaria.html

[29] CDC. How to report a case of malaria. Atlanta, GA: US Department of Health and Human Services, CDC; 2019. https://www.cdc.gov/malaria/report.html

[30] CDC. Malaria case surveillance report. Atlanta, GA: US Department of Health and Human Services, CDC; 2019. https://www.cdc.gov/malaria/resources/pdf/report/malaria_form.pdf

[31] CDC. National Notifiable Diseases Surveillance System (NNDSS): message validation, processing, and provisioning system. Atlanta, GA: CDC; 2022. https://www.cdc.gov/nndss/trc/data-systems/mvps.html#:~:text=The%20Message%20Validation%2C%20Processing%2C%20and,for%20their%20national%20surveillance%20efforts

[32] CDC. Malaria: treatment of malaria: guidelines for clinicians (United States). Atlanta, GA: US Department of Health and Human Services, CDC; 2020. https://www.cdc.gov/malaria/diagnosis_treatment/clinicians1.html

[33] World Health Organization. WHO malaria terminology, 2021 update. Geneva, Switzerland: World Health Organization; 2021. https://www.who.int/publications/i/item/9789240038400

[34] Brunette GW, Nemhauser JB, eds. CDC yellow book 2020: health information for international travel. New York, NY: Oxford University Press; 2017.

[35] Imwong M, Snounou G, Pukrittayakamee S, Relapses of Plasmodium vivax infection usually result from activation of heterologous hypnozoites. J Infect Dis 2007;195:927–33. 10.1086/512241 17330781

[36] Griffith KS, Lewis LS, Mali S, Parise ME. Treatment of malaria in the United States: a systematic review. JAMA 2007;297:2264–77. 10.1001/jama.297.20.2264 17519416

[37] World Health Organization. Guidelines for the treatment of malaria. 3rd ed. Geneva, Switzerland: World Health Organization; 2015. https://apps.who.int/iris/bitstream/handle/10665/162441/9789241549127_eng.pdf

[38] Barnett ED, MacPherson DW, Stauffer WM, The visiting friends or relatives traveler in the 21st century: time for a new definition. J Travel Med 2010;17:163–70. 10.1111/j.1708-8305.2010.00411.x 20536884

[39] Arguin PM. A definition that includes first and second generation immigrants returning to their countries of origin to visit friends and relatives still makes sense to me. J Travel Med 2010;17:147–9. 10.1111/j.1708-8305.2010.00412.x 20536881

[40] CDC. Blood specimens—specimen processing. Atlanta, GA: US Department of Health and Human Services, CDC; 2020. https://www.cdc.gov/dpdx/diagnosticprocedures/blood/specimenproc.html

[41] CDC. Staining for malaria parasites. Atlanta, GA: US Department of Health and Human Services, CDC; 2013. https://www.cdc.gov/dpdx/resources/pdf/benchAids/malaria/malaria_staining_benchaid.pdf

[42] DiMaio MA, Pereira IT, George TI, Banaei N. Performance of BinaxNOW for diagnosis of malaria in a US hospital. J Clin Microbiol 2012;50:2877–80. 10.1128/JCM.01013-12 22718936 PMC3421790

[43] CDC. Notice to readers: malaria rapid diagnostic test. Atlanta, GA: US Department of Health and Human Services, CDC. https://www.cdc.gov/mmwr/preview/mmwrhtml/mm5627a4.htm

[44] Cullen KA, Arguin PM; CDC. Malaria surveillance—United States, 2012. MMWR Surveill Summ 2014;63(No. SS-12):1–22. 25474160

[45] Bacon DJ, McCollum AM, Griffing SM, Dynamics of malaria drug resistance patterns in the Amazon basin region following changes in Peruvian national treatment policy for uncomplicated malaria. Antimicrob Agents Chemother 2009;53:2042–51. 10.1128/AAC.01677-08 19258269 PMC2681566

[46] Korsinczky M, Chen N, Kotecka B, Saul A, Rieckmann, Cheng Q Mutations in Plasmodium falciparum cytochrome b that are associated with atovaquone resistance are located at a putative drug-binding site. Antimicrob Agents Chemother 2000;44:2100–8. 10.1128/AAC.44.8.2100-2108.2000 10898682 PMC90020

[47] Price RN, Uhlemann AC, Brockman A, Mefloquine resistance in Plasmodium falciparum and increased pfmdr1 gene copy number. Lancet 2004;364:438–47. 10.1016/S0140-6736(04)16767-6 15288742 PMC4337987

[48] Ariey F, Witkowski B, Amaratunga C, A molecular marker of artemisinin-resistant Plasmodium falciparum malaria. Nature 2014;505:50–5. 10.1038/nature12876 24352242 PMC5007947

[49] Talundzic E, Okoth SA, Congpuong K, Selection and spread of artemisinin-resistant alleles in Thailand prior to the global artemisinin resistance containment campaign. PLoS Pathog 2015;11:e1004789. 10.1371/journal.ppat.1004789 25836766 PMC4383523

[50] World Health Organization. Report on antimalarial drug efficacy, resistance and response: 10 years of surveillance (2010–2019). Geneva, Switzerland: World Health Organization; 2020. https://www.who.int/publications/i/item/9789240012813

[51] Mace KE, Lucchi NW, Tan KR. Malaria Surveillance—United States, 2017. MMWR Surveill Summ 2021;70(No. SS-2):1–35. 10.15585/mmwr.ss7002a1 33735166 PMC8017932

[52] CDC. Simian malaria in a U.S. traveler—New York, 2008. MMWR Morb Mortal Wkly Rep 2009;58:229–32. 19282815

[53] Mali S, Steele S, Slutsker L, Arguin PM; CDC. Malaria surveillance-—United States, 2008. MMWR Surveill Summ 2010;59(No. SS-7):1–15. 20577158

[54] Ballard SB, Salinger A, Arguin PM, Desai M, Tan KR. Recommendations for using artemether-lumefantrine for the treatment of uncomplicated malaria in pregnant women in the United States. MMWR Morb Mortal Wkly Rep 2018;67:424–31. 10.15585/mmwr.mm6714a4 29649190 PMC5898222

[55] Mace KE, Arguin PM, Tan KR. Malaria surveillance-—United States, 2015. MMWR Surveill Summ 2018;67(No. SS-7):1–28. 10.15585/mmwr.ss6707a1 29723168 PMC5933858

[56] Kim HC, Pacha LA, Lee WJ, Malaria in the Republic of Korea, 1993–2007. Variables related to re-emergence and persistence of Plasmodium vivax among Korean populations and US forces in Korea. Mil Med 2009;174:762–9. 10.7205/MILMED-D-01-6208 19685850

[57] Vento TJ, Cardile AP, Littell CT, Compliance with malaria preventive measures by US military personnel deployed in support of Ebola control efforts in Liberia. San Diego, CA: Open Forum Infectious Diseases; 2015. https://academic.oup.com/ofid/article/2/suppl_1/1609/2634209

[58] Woods VM. Biggest threat to US troops in Liberia is malaria, not Ebola. Washington, DC: US Army; 2014. https://www.army.mil/article/139340/biggest_threat_to_u_s_troops_in_liberia_is_malaria_not_ebola

[59] CDC. Insect repellents help prevent malaria and other diseases spread by mosquitoes. Atlanta, GA: US Department of Health and Human Services, CDC; 2015. https://www.cdc.gov/malaria/resources/pdf/fsp/repellents_2015.pdf

[60] Francis BC, Gonzalo X, Duggineni S, Epidemiology and clinical features of imported malaria in East London. New York, NY: Journal of Travel Medicine; 2016. https://academic.oup.com/jtm/article/23/6/taw060/2751000 10.1093/jtm/taw060 27601534

[61] Tatem AJ, Jia P, Ordanovich D, The geography of imported malaria to non-endemic countries: a meta-analysis of nationally reported statistics. Lancet Infect Dis 2017;17:98–107. 10.1016/S1473-3099(16)30326-7 27777030 PMC5392593

[62] Mischlinger J, Rönnberg C, Álvarez-Martínez MJ, Imported malaria in countries where malaria is not endemic: a comparison of semi-immune and nonimmune travelers. Clin Microbiol Rev 2020;33:e00104-19. 10.1128/CMR.00104-19 32161068 PMC7067581

[63] de Gier B, Suryapranata FST, Croughs M, Increase in imported malaria in the Netherlands in asylum seekers and VFR travellers. Malar J 2017;16:60. 10.1186/s12936-017-1711-5 28148300 PMC5288937

[64] Cohen NJ, Brown CM, Alvarado-Ramy F, Travel and border health measures to prevent the international spread of Ebola. MMWR Suppl 2016;65(No. Suppl 3):57–67. 10.15585/mmwr.su6503a9 27390092

[65] Mace KE, Arguin PM, Lucchi NW, Tan KR. Malaria surveillance—United States, 2016. MMWR Surveill Summ 2019;68(No. SS-5):1–35. 10.15585/mmwr.ss6805a1 31099769

[66] Cullen KA, Mace KE, Arguin PM; CDC. Malaria surveillance—United States, 2013. MMWR Surveill Summ 2016;65(No. SS-2):1–22. 10.15585/mmwr.ss6502a1 26938139

[67] Mace KE, Arguin PM. Malaria surveillance—United States, 2014. MMWR Surveill Summ 2017;66(No. SS-12):1–24. 10.15585/mmwr.ss6612a1 28542123 PMC5829864

[68] Scott LA, Dunlop SJ, Walz EJ, Prescription drug-dispensing limits in the USA—implications for malaria chemoprophylaxis among VFR travelers. J Travel Med 2018;25:tay039. 10.1093/jtm/tay039 29893891 PMC6676974

[69] Walz EJ, Volkman HR, Adedimeji AA, Barriers to malaria prevention in US-based travellers visiting friends and relatives abroad: a qualitative study of West African immigrant travellers. J Travel Med 2019;26:tay163. 10.1093/jtm/tay163 30602033 PMC6679970

[70] Walz EJ, Wanduragala D, Adedimeji AA, Community-based participatory research in travel medicine to identify barriers to preventing malaria in VFR travellers. J Travel Med 2019;26:tay148. 10.1093/jtm/tay148 30535124 PMC6628254

[71] Volkman HR, Walz EJ, Wanduragala D, Barriers to malaria prevention among immigrant travelers in the United States who visit friends and relatives in sub-Saharan Africa: a cross–sectional, multi-setting survey of knowledge, attitudes, and practices. PLoS One 2020;15:e0229565. 10.1371/journal.pone.0229565 32163426 PMC7067457

[72] Frosch AE, Thielen BK, Alpern JD, Antimalarial chemoprophylaxis and treatment in the USA: limited access and extreme price variability. J Travel Med 2021;taab117. 10.1093/jtm/taab117 34343310 PMC9282095

[73] Schultz JS, Atherly AJ, Henao-Martínez AF. A deadly wait for US health insurance coverage—sitting on the couch with malaria. Am J Trop Med Hyg 2018;99:24–6. 10.4269/ajtmh.18-0010 29761755 PMC6085814

[74] Freedman DO. Tafenoquine: integrating a new drug for malaria prophylaxis into travel medicine practice. J Travel Med 2019;26:tay140. 10.1093/jtm/tay140 30496585

[75] Tan KR, Hwang J. Tafenoquine receives regulatory approval in USA for prophylaxis of malaria and radical cure of Plasmodium vivax. J Travel Med 2018;25:tay071. 10.1093/jtm/tay071 30137454 PMC10956546

[76] Buss I, Genton B, D’Acremont V. Aetiology of fever in returning travellers and migrants: a systematic review and meta-analysis. J Travel Med 2020;27:taaa207. 10.1093/jtm/taaa207 33146395 PMC7665639

[77] Camprubí-Ferrer D, Cobuccio L, Van Den Broucke S, Causes of fever in returning travelers: a European multicenter prospective cohort study. J Travel Med 2022;29:taac002. 10.1093/jtm/taac002 35040473

[78] CDC. Malaria diagnosis (United States). Atlanta, GA: US Department of Health and Human Services, CDC; 2018. https://www.cdc.gov/malaria/diagnosis_treatment/diagnosis.html

[79] Abanyie FA, Arguin PM, Gutman J. State of malaria diagnostic testing at clinical laboratories in the United States, 2010: a nationwide survey. Malar J 2011;10:340. 10.1186/1475-2875-10-340 22074250 PMC3225402

[80] Prestel C, Tan KR, Abanyie F, Jerris R, Gutman JR. Malaria diagnostic practices in US laboratories in 2017. J Clin Microbiol 2018;56:e00461–18. 10.1128/JCM.00461-18 29875196 PMC6062816

[81] CDC. DPDx—laboratory identification of parasites of public health concern: diagnostic assistance. Atlanta, GA: US Department of Health and Human Services, CDC; 2022. https://www.cdc.gov/dpdx/dxassistance.html

[82] CDC. DPDx—laboratory identification of parasites of public health concern: training. Atlanta, GA: US Department of Health and Human Services, CDC; 2019. https://www.cdc.gov/dpdx/training.html

[83] World Health Organization. Statement by the Malaria Policy Advisory Group on the urgent need to address the high prevalence of pfhrp2/3 gene deletions in the Horn of Africa and beyond. Geneva, Switzerland: World Health Organization; 2021. https://www.who.int/news/item/28-05-2021-statement-by-the-malaria-policy-advisory-group-on-the-urgent-need-to-address-the-high-prevalence-of-pfhrp2-3-gene-deletions-in-the-horn-of-africa-and-beyond

[84] CDC. Quinidine availability in the United States. Atlanta, GA: US Department of Health and Human Services, CDC; 2017. https://www.cdc.gov/malaria/new_info/2017/Quinidine_2017.html

[85] Rosenthal PJ, Tan KR. Expanded availability of intravenous artesunate for the treatment of severe malaria in the United States. Am J Trop Med Hyg 2019;100:1295–6. 10.4269/ajtmh.19-0230 30927931 PMC6553912

[86] Food and Drug Administration. FDA approves only drug in US to treat severe malaria. Silver Spring, MD: US Department of Health and Human Services, Food and Drug Administration; 2020. https://www.fda.gov/news-events/press-announcements/fda-approves-only-drug-us-treat-severe-malaria

[87] Amivas. Artesunate for injection. Frederick, MD: Amivas; 2021. https://amivas.com

[88] CDC. Intravenous artesunate for treatment of severe malaria in the United States. Atlanta, GA: US Department of Health and Human Services, CDC; 2021. https://www.cdc.gov/malaria/diagnosis_treatment/artesunate.html

[89] Cullen KA, Arguin PM; CDC. Malaria surveillance—United States, 2011. MMWR Surveill Summ 2013;62(No. SS 5):1–17. 24172939

[90] Food and Drug Administration. Revised recommendations to reduce the risk of transfusion-transmitted malaria: guidance for industry. Silver Spring, MD: US Department of Health and Human Services, Food and Drug Administration; 2020. https://www.fda.gov/regulatory-information/search-fda-guidance-documents/revised-recommendations-reduce-risk-transfusion-transmitted-malaria

[91] Health Resources & Services Administration. Donation and transplantation statistics. Rockville, MD: US Department of Health and Human Services, Health Resources & Services Administration; 2021. https://bloodstemcell.hrsa.gov/data/donation-and-transplantation-statistics

[92] Global Malaria Programme. Intermittent screening and treatment in pregnancy and the safety of ACTs in the first trimester. Geneva, Switzerland: World Health Organization; 2015. https://apps.who.int/iris/handle/10665/338496?locale-attribute=es&

[93] Andrejko KL, Mayer RC, Kovacs S, The safety of atovaquone-proguanil for the prevention and treatment of malaria in pregnancy: a systematic review. Travel Med Infect Dis 2019;27:20–6. 10.1016/j.tmaid.2019.01.008 30654041 PMC9074802

[94] Mayer RC, Tan KR, Gutman JR. Safety of atovaquone-proguanil during pregnancy. J Travel Med 2019;26:tay138. 10.1093/jtm/tay138 30544231 PMC6590067

[95] Levine RS, Peterson AT, Benedict MQ. Distribution of members of Anopheles quadrimaculatus say s.l. (Diptera: Culicidae) and implications for their roles in malaria transmission in the United States. J Med Entomol 2004;41:607–13. 10.1603/0022-2585-41.4.607 15311451

[96] CDC. Data modernization initiative: an urgent need to modernize. Atlanta, GA: US Department of Health and Human Services, CDC; 2022. https://www.cdc.gov/surveillance/surveillance-data-strategies/dmi-investments.html

